# Supplementary material for: Qualitative process study to explore the perceived burdens and benefits of a digital intervention for self-managing high blood pressure in Primary Care in the UK
Source: BMJ Open. 2018 May 8;8(5):e020843. doi: 10.1136/bmjopen-2017-020843 (PMC5942415; doi:10.1136/bmjopen-2017-020843)
Supplement: Supplementary file 2 [file bmjopen-2017-020843supp002.pdf]

Supplementary File 2. Interview schedules for intervention and usual care group

Intervention group interview schedule

<u>Opening questions</u>

Can you tell me about what it is like for you to have high blood pressure?

How did you feel when you were first told you had high blood pressure?

Can you tell me why you decided to sign up for the Home BP study?

I'm really interested in hearing about your experiences of using HOME BP, can you tell me all about it?

Can you tell me about anything that you have liked about the HOME BP study so far?

Can you tell me about anything that you have disliked?

Can you tell me about any advantages of using HOME BP?

And can you tell me about any disadvantages of using HOME BP?

How do you feel about your high blood pressure now?

Do you think you would choose to keep on using the HOME BP programme if it was still available after a year?

Why/ Why not?

Would you recommend the HOME BP programme to other people with high blood pressure?

<u>Self-monitoring</u>

<u>Experiences of monitoring:</u>

Can you tell me about what it was like when you were learning how to use your BP monitor at home?

Can you tell me about the first time you used your BP monitor at home in the study?

Can you tell me about what it has been like to monitor your blood pressure yourself?

How did it make you feel?

Had you ever monitored your own blood pressure before you took part in the HOME BP study?

If yes:

Can you tell me a bit more about this?

Can you tell me whether you've noticed any changes in how you monitor your blood pressure since you've started the HOME BP programme?

All: Can you tell me about any concerns that you have about monitoring your blood pressure?

Adherence to the monitoring schedule:

The HOME BP programme reminds you to monitor your blood pressure for 7 days every 4 weeks. Can you tell me about what it has been like for you to try and monitor your blood pressure for 7 days every 4 weeks?

Can you tell me about the time of day you have been monitoring your blood pressure so far?

How do you feel about the instructions HOME BP gives about when to monitor?

Was there anything that helped you to monitor your own blood pressure?

Was there anything that made it harder to monitor your own blood pressure?

Can you tell me about any time when you had to skip your blood pressure monitoring or change when you did it in the day?

Can you tell me how you found it entering your readings on the HOME BP programme?

Feedback messages

Remember after you enter your readings on HOME BP, you see a message which says ‘your readings were amber, or green’? What did you think about these messages?

Can you tell me about how you felt when you saw a message about your blood pressure readings on HOME BP?

How did you feel about monitoring your own blood pressure after reading that message?

Did you have any very high or very low readings (red/blue) when you were monitoring in the HOME BP study? How did you feel?

2. Medication change

Can you tell me about what it’s like for you taking medication for your high blood pressure?

Have you been recommended to make a medication change during the HOME BP study?

If yes:

How did you feel about making a change to your medication when HOME BP recommended it?

Did you make this change to your medication?

If they chose to make a change:

Can you tell me about what it was like to make a medication change in the HOME BP study?

Can you tell me about how the medication change came about?

How did you get on with your new medication?

How would you feel if HOME BP recommended a medication change for you now?

If they chose not to make a change –

Can you tell me about why you decided not to change your medication?

How would you feel if HOME BP recommended another medication change for you now?

If no:

If patient has **not** *been recommended a med change:* How do you feel about changing your medication if your blood pressure stayed too high for too long?

Can you tell me about any concerns that you have about doing this?

All:

How did you feel about making changes to your medication before you took part in HOME BP?

3. HCP support

Can you tell me about the contact you have had with your GP or nurse so far in the HOME BP study?

Can you tell me about how you have found the support from your GP or nurse while you were taking part in the HOME BP study?

Can you tell me what you thought about receiving emails from your nurse about HOME BP?

If needed, follow-up questions:

Have you seen your GP about your blood pressure since the start of the study?

And what about appointments with the nurse?

Can you tell me about how you have found the emails from HOME BP?

4. Healthy changes

Can you tell me about anything else that you do to try and manage your high blood pressure?

There is an option on the HOME BP programme to look at online sessions about making healthy changes that can help lower your blood pressure, I wonder if you remember if you have seen it?

If no:

Can you tell about how you might feel about making healthy changes to help lower your blood pressure?

If yes:

Can you tell me about how you found the session on healthy changes?

How did you feel about making the healthy changes this session talked about?

If they chose a change: Can you tell me about what made you choose this/these ones?

How did you find the online session about xxxx (cutting down on salt/alcohol/eating a healthier diet/doing more physical activity/ losing weight)?

Can you tell me about what it was like for you trying out this healthy change?

How did making a healthy change like this make you feel?

<u>*Or if they didn't choose any*</u>*:* Can you tell me about why you didn't want to choose a healthy change?

All: Can you tell me about any concerns that you have about making a new healthy change?

Is there anything else you would like to tell me about the HOME BP study that we haven't already talked about?

Usual care group interview schedule

General questions about BP

Can you tell me about what it is like for you to have high blood pressure?

How did you feel when you were first told you had high blood pressure?

Can you talk me through what (if anything) you currently do to manage/control your high blood pressure?

Can you tell me about how your GP and practice nurse are involved in helping you to control your high blood pressure?

How do you feel about monitoring your own blood pressure at home?

If they already monitor – can you tell me a bit more about this?

Can you tell me about what it's like for you taking medication for your high blood pressure?

Can you tell me how you feel about your GP making changes to your blood pressure medication?

HOME BP study experience

Can you tell me why you signed up for the Home BP study?

Can you tell me all about what it has been like for you being in the HOME BP study in the usual care group?

At the start of the HOME BP study, you had a baseline appointment with a nurse at your GP Practice – can you tell me about that?

How did you find it using the HOME BP programme to fill in your questionnaires online at the start of the study?

After you had completed your questionnaires, did you have an appointment with your GP to check your blood pressure medication? Can you tell me about this?

HCP support

Can you tell me about the contact you have had with your GP or nurse so far in the HOME BP study?

Can you tell me about how you have found the support from your GP or nurse while you were taking part in the HOME BP study?
