# Supplementary material for: Qualitative process study to explore the perceived burdens and benefits of a digital intervention for self-managing high blood pressure in Primary Care in the UK
Source: BMJ Open. 2018 May 8;8(5):e020843. doi: 10.1136/bmjopen-2017-020843 (PMC5942415; doi:10.1136/bmjopen-2017-020843)
Supplement: Author's manuscript [file bmjopen-2017-020843.draft_revisions.pdf]

BMJ Open

BMJ Open is committed to open peer review. As part of this commitment we make the peer review history of every article we publish publicly available.

When an article is published we post the peer reviewers' comments and the authors' responses online. We also post the versions of the paper that were used during peer review. These are the versions that the peer review comments apply to.

The versions of the paper that follow are the versions that were submitted during the peer review process. They are not the versions of record or the final published versions. They should not be cited or distributed as the published version of this manuscript.

BMJ Open is an open access journal and the full, final, typeset and author-corrected version of record of the manuscript is available on our site with no access controls, subscription charges or pay-per-view fees (http://bmjopen.bmj.com).

If you have any questions on BMJ Open's open peer review process please email info.bmjopen@bmj.com

BMJ Open

Perceived burdens and benefits of self-management interventions: A qualitative process study of an online intervention for self-managing high blood pressure.

Journal:	*BMJ Open*
Manuscript ID	bmjopen-2017-020843
Article Type:	Research
Date Submitted by the Author:	27-Nov-2017

Complete List of Authors:	Morton, Katherine; University of Southampton, Psychology<br>Dennison, Laura; University of Southampton, Psychology<br>Bradbury, Katherine; University of Southampton, Psychology<br>Band, Rebecca; University of Southampton, Psychology<br>May, Carl; University of Southampton, Faculty of Health Sciences<br>Raftery, James; Southampton University, School of Medicine<br>Little, Paul; University of Southampton, Primary Care and Population Science<br>McManus, Richard; University of Oxford, Dept of Primary Care Health Sciences<br>Yardley, Lucy ; University of Oxford, Nuffield Department of Primary Health Care Sciences

Keywords:	Self-management, digital intervention, qualitative, treatment burden

SCHOLARONE™
Manuscripts

For peer review only

Title page

Perceived burdens and benefits of self-management interventions: A qualitative process study of an online intervention for self-managing high blood pressure.

Kate Morton[a]*, Laura Dennison[a], Katherine Bradbury[a], Rebecca Band[a], Carl May[b], James Raftery[c], Paul Little[d], Richard J. McManus[e], & Lucy Yardley[a]

a. Academic unit of psychology, University of Southampton, Southampton, UK
b. Faculty of Health Sciences, University of Southampton, Southampton, UK
c. Faculty of Medicine, University of Southampton, Southampton, UK
d. Primary Care Research, University of Southampton, Southampton, UK
e. Nuffield Department of Primary Care Health Sciences, University of Oxford, Oxford, UK

*Corresponding author: ksm1r13@soton.ac.uk; 02380 592234

Room 3063, B44, University of Southampton, Southampton, SO17 1BJ

Word count 4888

For peer review only - http://bmjopen.bmj.com/site/about/guidelines.xhtml

ABSTRACT

Objectives: Digital health interventions can change patients' experiences of managing their health, either creating additional burden or improving their experience of healthcare. This qualitative study aims to explore perceived burdens and benefits for patients using an online self-management intervention for high blood pressure. A secondary aim is to further our understanding of how best to capture these outcomes when evaluating health interventions.

Design: Inductive qualitative process study nested in a randomised controlled trial.

Setting: Primary Care in the UK

Participants: 35 participants taking antihypertensive medication and with uncontrolled blood pressure at baseline took part in semi-structured telephone interviews.

Intervention: Online self-management intervention to support blood pressure self-monitoring and medication change when recommended by the healthcare professional.

Analysis: Data were analysed using inductive thematic analysis with techniques from grounded theory.

Results: Seven themes were developed which reflected perceived burdens and benefits of using the intervention, including worry about health, uncertainty about self-monitoring, and reassurance. A model was developed to show how beliefs about their condition and treatment appeared to influence participants' appraisal of the value of the intervention, suggesting that considering illness and treatment perceptions in Burden of Treatment theory could further our understanding of how individuals appraise the personal costs and benefits of self-managing their health.

For peer review only

For peer review only - http://bmjopen.bmj.com/site/about/guidelines.xhtml

Conclusions: Patients' appraisal of the burden or benefit of using a complex self-management intervention seemed to be influenced by both experiences within the intervention (such as perceived availability of support) and beliefs about their condition and treatment (such as perceived control and risk of side effects). Developing our ability to adequately capture these salient burdens and benefits for patients could help enhance evaluation of self-management interventions in future. Many participants perceived important benefits from using the intervention, highlighting the need for theory to allow that engaging in self-management can include positive as well as negative aspects.

Trial registration: ISRCTN13790648. Registered 14 May 2015.

Keywords: Self-management; digital intervention; qualitative; treatment burden

Strengths and limitations of this study:

- The exploratory, open approach to data collection enabled us to capture whichever benefits or burdens were most salient to the participants.
- This inductive approach highlighted some novel reactions to using self-management digital interventions.
- We only interviewed participants at one point in time, so were unable to gain an understanding of dynamic changes in perceived benefits or burdens over time.
- Both well and poorly controlled hypertensive patients took part in the interviews, but it was difficult to recruit low users of the intervention which could limit the generalisability of the findings.
- Qualitative data is not commonly used in health economics evaluation, so further work would be needed to understand how relevant outcomes could best be captured quantitatively.

For peer review only

For peer review only - http://bmjopen.bmj.com/site/about/guidelines.xhtml

BACKGROUND

The work involved in looking after one's health when living with a chronic condition can include complex tasks such as organising and adhering to treatment regimes, interacting with healthcare professionals (HCPs), regular monitoring of health indicators, and making health-related decisions, all of which can accumulate into a considerable burden for people[1]. Digital self-management interventions are often developed to improve health outcomes, but these interventions could also either increase or minimise the burden of the process of healthcare for patients. Developing our understanding of the burdens of self-management can help to better optimise the delivery of healthcare to improve adherence and well-being[1-3]. Burden of Treatment (BoT) theory provides a mechanism for understanding these experiences in the context of patients' personal capacity to cope, with emphasis on the role of wider healthcare systems and social networks available to the patient[1].

Health economic evaluations also focus on understanding the impact of healthcare on patients, seeking to weigh up the resources used against the health outcomes in order to better inform decision-making. Recent guidelines for economic evaluations in health and medicine recommend adopting a societal perspective such that all relevant outcomes are evaluated, rather than focusing only on formal healthcare costs[4]. In particular, personal costs such as time spent in self-care should be included. Consequently, BoT theory and health economic evaluations share an interest in adequately capturing the wider burdens or personal costs of engaging with healthcare. For consistency in terminology in this paper, negative outcomes/personal costs of healthcare will be referred to as 'burdens'.

BoT theory considers patients' time spent on healthcare as a resource that is used by the healthcare system, while health economic evaluation counts time as an 'opportunity cost' whereby the patient 'spends' time that could have been spent on something other than healthcare. However, subjective experiences of time spent on digital interventions may be varied and complex. Heterogeneity in the

For peer review only - http://bmjopen.bmj.com/site/about/guidelines.xhtml

relative value placed on the outcomes of the intervention[5] may mean that for some participants the time spent engaging with elements of an intervention is not perceived as a burden but rather as a benefit, either because it is interesting, pleasant or meaningful in and of itself or because of the positive outcomes it can lead to. In other words, some people may actually like engaging with healthcare. The value of exploring the personal benefits of intervention participation has not received as much focus as understanding the costs, such as treatment burden. McNamee et al.[6] propose that the health research guidelines for economic analysis may need to be adjusted for digital health interventions to ensure we can fully capture the heterogeneous costs and benefits arising when complex interventions are implemented in complex systems.

To further our understanding of how patients perceive benefits and burdens when using digital health interventions, we carried out a qualitative process study. The online HOME BP intervention was developed based on best practice recommendations to help improve hypertension in poorly controlled patients by facilitating self-monitoring of blood pressure (BP) at home and prompting appropriate intensification of medication by healthcare professionals[7]. This intervention could help to minimise the treatment burden of hypertension by providing a healthcare system in which HCPs have sight of patients' home readings, streamlining the process for finding the most effective medication without the need for attending the GP Practice. However, HOME BP is a complex, interactive multi-component intervention, which creates potential diversity in the perceived burden and benefits for participants using it. The contexts in which the intervention is embedded may also be diverse, and factors such as individual differences in patients' health status, beliefs about medication and risks of high BP, availability of time and resources, and access to support may influence how the intervention is perceived and valued. The HOME BP intervention was developed using the person-based approach[8] which emphasises the importance of understanding participants' unique perspectives and different situations when developing and implementing digital interventions. Adopting a more granular approach to the evaluation of benefit and burden is

For peer review only - http://bmjopen.bmj.com/site/about/guidelines.xhtml

consistent with the person-based approach, and with the BoT approach of fully understanding the participants' perspective.

The present study aimed to explore the perceived burden and benefits of using a digital health intervention for self-managing BP using qualitative process interviews with intervention and usual care participants taking part in a randomised controlled trial (RCT).This paper seeks to interpret these findings in terms of the implications for optimising the capture of perceived costs and benefits in health economic evaluations and evaluating the burden of treatment.

METHODS

Design

A qualitative process study embedded in the Home BP trial[7] was approved by the University of Southampton and NHS Research Ethics committees. The COREQ checklist (Consolidated criteria for reporting qualitative studies) was used to ensure comprehensive reporting of the study[9] (supplementary file 1).

Intervention

The HOME BP online programme supports participants to self-manage their high BP, primarily via home self-monitoring of BP and making changes to dose/drug type when recommended by the HCP. Lifestyle change modules are also available, but optional. Participants using HOME BP were supported by a 'prescriber' (GP or nurse prescriber responsible for changing medication) and a 'supporter' (nurse or healthcare assistant who supported participants in self-monitoring and choosing lifestyle changes). Table 1 describes the HOME BP intervention in more detail.

For peer review only

For peer review only - http://bmjopen.bmj.com/site/about/guidelines.xhtml

Table 1 HOME BP Intervention Characteristics

Target behaviour	Description
Self-monitoring BP	Participants monitor their BP at home for 7 days every 4 weeks. After 7 days, they enter their BP readings into the HOME BP online programme and receive instant automated feedback using a traffic light system. If BP is very high (red) or very low (blue), they are told to contact their GP Practice. If BP is above target (amber), they are told their prescriber will contact them about a medication change. If BP is on target (green), they are congratulated and asked to monitor their BP again next time.
Medication change	The prescriber plans three potential medication changes with the participant at the start of the study. Prescribers are informed by email when a medication change is recommended and can implement a pre-planned change without needing to see the participant for an appointment.
Optional lifestyle changes	Nine weeks after randomisation, participants have the option of choosing an online session to support lifestyle change to help control their BP, specifically weight management, salt reduction, healthy diet, physical activity, or alcohol reduction. Participants are alerted by email when this becomes available, and see an option to view the healthy lifestyles session each time they log on to HOME BP. The online lifestyle change sessions can be started at any time during the 12-month trial, after nine weeks.

For peer review only

For peer review only - http://bmjopen.bmj.com/site/about/guidelines.xhtml

Participants

Patients were eligible to take part in the HOME BP trial if they had uncontrolled hypertension (mean BP reading of 140/90 mm Hg or more at baseline) managed in Primary Care, were prescribed 1-3 antihypertensive medications at baseline, and aged over 18 (see[7] for full inclusion and exclusion criteria).

Both intervention and usual care participants were invited to take part in interviews as we felt that obtaining an understanding of managing BP in usual care would aid interpretation of the perceived burden and benefits of the intervention. We aimed to speak to participants at a range of time-points during the 12-month trial from 10 weeks onwards as this gave participants the opportunity to become familiar with HOME BP. No new intervention content was introduced after nine weeks.

Recruitment and interview procedure

A sub-sample of RCT participants were invited by email to provide feedback on their experiences of managing their BP (n=78). Informed consent was taken by post or online, depending on participant preference. Recruitment was initially opportunistic, but subsequently a purposive approach was adopted to target younger participants, low engagers, and those with recent uncontrolled self-monitored BP readings, informed by the concurrent analysis. Recruitment was stopped once the researchers agreed that data saturation had been reached and no new burdens or benefits were arising.

Semi-structured interview schedules were co-developed by experts in health psychology (KM, KB, RB, LY, LD), health economics (JR) and sociology (CM). Open, inductive questions were carefully selected to elicit data about the burden and benefits of BP management perceived as most salient by the participants (see Supplementary File 2 for interview schedules). The interviews were conducted by telephone to minimise the burden on participants, except in one case where the participant asked to meet face-to-face due to struggling with hearing on the telephone. The interviews took place

For peer review only

For peer review only - http://bmjopen.bmj.com/site/about/guidelines.xhtml

between February 2016 and February 2017. Each participant was given a £10 gift voucher to thank them for their time.

All interviews were conducted by KM (MSc, BSc. Termed "the researcher"), a female PhD candidate in Health Psychology who was also employed as a research assistant. Each interview was audio-recorded, and the researcher also took notes and completed a self-reflection log afterwards to record any emerging thoughts on the data. Audio-recordings were transcribed verbatim and checked thoroughly by the researcher.

Analysis

The analysis was an iterative process led by KM, supported by frequent discussion of emerging themes with LY and LD (who have extensive experience in qualitative research) along with input regarding health economic and sociological perspectives (JR and CM). Inductive thematic analysis methods were used[10 11] with techniques from grounded theory such as memoing, constant comparison, and diagramming to enhance our understanding and facilitate the development of higher themes[12 13]. Data collection and analysis ran concurrently to enable purposive sampling based on analytic insights. Thorough line-by-line coding was undertaken in NVivo 10[14], and a coding manual was developed which evolved as more data were collected and coded. The emerging codes were constantly checked against the raw data to ensure the analysis was driven by the participants' own language and experiences.

All data relating to burdens and benefits of managing BP were analysed. We also coded factors that appeared to influence perceptions of burdens and benefits to facilitate an in-depth understanding of how participants appraised the intervention's value. A broad and open definition was adopted whereby benefits and burdens were defined as positive and negative outcomes or experiences of engaging in the intervention[15], in order to facilitate a comprehensive representation of all potentially relevant data.

For peer review only

For peer review only - http://bmjopen.bmj.com/site/about/guidelines.xhtml

RESULTS

Participant characteristics

28 of 54 participants from the intervention group (52%), and 7 of 24 usual care participants (29%) agreed to be interviewed. Most participants who did not take part chose not to reply, but those who did said they did not have anything to report on the trial (n = 3 in usual care). The participants were from 19 different GP Practices. Table 2 shows the sociodemographic and intervention details of the sample.

For peer review only

Table 2. Sociodemographic and intervention participant data (n=35)

	Intervention participants	Usual care participants
N	28	7
Average duration of interview (range)	39 (15-67) minutes	28 (22-40) minutes
Average age (range)	65 (41-87) years	67 (52-77) years
Gender	71% female	43% female
Ethnicity		
White	24	6
Black African	1	
Pakistani	1	
Other	2	1
Education levels	9 No formal education	2 No formal education

For peer review only - http://bmjopen.bmj.com/site/about/guidelines.xhtml

	8 GCSE or A-level	3 GCSE or A-level
	10 Higher Education	1 Higher Education
	1 Other	1 Other
Number of weeks into study	23 (10-57) weeks	17 (7 to 24) weeks
Poorly controlled BP at the time of the interview	10/28 (36%)	N/A*
Medication change recommended during the study	15/28 (54%)	N/A
Accessed optional healthy lifestyles session	15/28 (54%)	N/A

*As BP self-monitoring was a key component of the intervention, BP readings were available for the intervention group throughout the duration of the study but data about BP from the usual care group were only available at RCT baseline and follow-up points.

Themes

Table 3 presents seven themes exploring perceptions of burdens and benefits of the HOME BP intervention. One meta-theme also emerged concerning how illness and treatment beliefs about high BP appeared to influence participants' perceptions about the intervention's burdens and benefits, and this is discussed in relation to each theme it applies to. Figure 1 shows how illness and treatment perceptions about BP appeared to relate to the sub-themes identified by the thematic analysis.

For peer review only

For peer review only - http://bmjopen.bmj.com/site/about/guidelines.xhtml

208 Table 3. Themes and sub-themes relating to perceived burdens and benefits of the intervention

Themes	Sub-themes	Exemplar participant quote
Benefit of reassurance from seeing BP readings	Reassurance when BP readings are well-controlled	"I'm so pleased. And my mind is at rest when we go on holidays and all that...I'm alright. I'm alright sort of thing. Yeah, peace of mind" (Intervention p9, well-controlled)
	Reassurance from keeping an eye on BP	"It made me much more aware of what the problem is with the high blood pressure and by monitoring it so regularly, I know exactly where I stand with it" (Intervention p15, well-controlled)
Benefit of motivation for lifestyle change from seeing BP readings	Seeing BP readings motivated lifestyle change	"It is quite interesting to see the effects of what I'm doing on the blood pressure and everything. So, I think that is – it is quite good" (Intervention p18, well-controlled)
Benefit of better	Perceived health improvements	"It helped me to change my medication and then because of change of medication,

For peer review only - http://bmjopen.bmj.com/site/about/guidelines.xhtml

health	from medication changes	my blood pressure went down. So definitely there is a benefit" (Intervention p16, well-controlled)
	Intervention can facilitate management of side effects	“That medication didn’t work, in that I was on holiday and my ankles swelled up so much – and my feet and my legs, so much so that I couldn’t see my toes. So I stopped taking that medication. Was called back to the GP. And I’m now on a medication that works for me and is managing the blood pressure” (Intervention p7, well-controlled)
Burden of worrying about health	Negative emotional responses to seeing high readings	"I was actually quite shocked because it was a—a lot higher" (Intervention p6, poorly controlled)
	Worrying about medication change affecting health	"I don’t want to get more medication ‘cause I’m already on a high dose and I don’t want to increase it because it worries me about my kidneys" (Intervention p24, poorly controlled)
Burden of uncertainty from self-monitoring	Uncertainty about whether readings are representative	"If someone only ever takes it in the morning, and you tend to get those lower readings, are you really getting a true picture of what they’re like in the afternoon or the evening?" (Intervention p10, well-controlled)

For peer review only - http://bmjopen.bmj.com/site/about/guidelines.xhtml

	Uncertainty about what to do about high or low readings	"I don't know what's going to happen in respect to that [amber feedback]. Whether I'm going to get a call from my GP, or whether he – so I'm a little bit, like, you know, in the air. I don't really know what's going to happen in that respect" (Intervention p22, poorly controlled)
Burden of thinking about making healthy lifestyle changes	Worry or guilt about not engaging with healthy changes	"I have looked at it [online healthy lifestyles session]. I wouldn't say I've looked at it seriously, and I need to" (Intervention p4, poorly controlled)
Burden of the practicalities of adhering to intervention procedures	Burden of fitting self-monitoring into the day	"I like to get up and have a cup of coffee and I'm thinking 'Well, let's get the blood pressure done first because otherwise I can't do that, you know, for a while afterwards.' So, I've found that quite—quite difficult" (Intervention p5, poorly controlled).

209

For peer review only - http://bmjopen.bmj.com/site/about/guidelines.xhtml

For peer review only

For peer review only - http://bmjopen.bmj.com/site/about/guidelines.xhtml

211 Insert Figure 1 here

212 Benefit of reassurance from seeing BP readings

213 *Reassurance when BP readings are well-controlled*

214 Seeing well-controlled readings when self-monitoring BP gave participants peace of mind which was
215 widely perceived as a benefit of the intervention. People described feeling relieved that their BP
216 readings were lower than at the GP Practice, and felt this gave them more insight into what their BP
217 was like most of the time.

218 "What I do like about it is taking the blood pressure here at home, the readings are lower. And I find
219 that quite reassuring that my blood pressure is not always high" (Intervention p11, well-controlled)

220 Several usual care participants had decided to use their own BP monitors independently of the
221 online intervention, and this group also described feeling reassurance when seeing their BP was
222 well-controlled.

223 *Reassurance from keeping an eye on BP*

224 Most participants liked having an increased focus on their BP through regular monitoring and found
225 it interesting to compare their readings over time. However one participant perceived that taking BP
226 regularly could encourage too much attention on your health, which was a potential burden of the
227 intervention for her (Intervention p28, BP control unknown as did not enter BP readings online). This
228 participant had low concern about her BP generally, and was not motivated to engage in self-
229 management.

230 Even when participants had poorly-controlled readings, many felt a benefit from the intervention as
231 it enabled them to regularly check their BP and detect any problems instantly rather than carrying
232 on unaware.

For peer review only - http://bmjopen.bmj.com/site/about/guidelines.xhtml

"I think it's helping me to know where my blood pressure stands because it's a regular thing every month" (Intervention p24, poorly-controlled)

The knowledge that home BP readings were shared with the prescriber reassured participants as they knew that any problems would not only be detected but also dealt with at the time, making them feel well cared for. This contrasted with the perceived burden of managing BP in usual care where some participants felt concerned that their GP did not change their medication when their home readings were too high, or would have liked more regular contact with their Practice to check their BP and medication.

"It would be nice to have it checked, I guess, you know, every three months or whatever. How—however often. I mean, how do they know that everything is working?" (Usual care p4).

This shows that although participants in usual care gained reassurance from seeing low readings when they monitored at home, the lack of interaction with the Practice could cause concern when readings were high or when patients did not regularly monitor BP at home of their own accord.

Benefit of motivation for lifestyle change from seeing BP readings

Some participants were motivated to increase their physical activity, engage in stress management activities or healthy eating because they could see this had a positive impact on their BP readings. This helped them feel more in control of their BP.

"By taking the readings regularly and frequently, it gave me more of a feedback straightaway if you like about anything, changes that I did make like a bit of exercise or…practicing relaxation and this sort of thing. So that was quite nice, it was nice to feel that I was more in control of it again" (Intervention p20, well-controlled)

For peer review only - http://bmjopen.bmj.com/site/about/guidelines.xhtml

Other participants felt frustrated after making lifestyle changes in the past which had no effect on their BP. This made them feel that lifestyle was ineffective for controlling BP.

"I'm a completely different person. My diet's completely different. And my blood pressure remained the same. So I've done literally everything you physically possibly can to help yourself, and nothing's worked" (Intervention p1, well-controlled).

Benefit of better health

Perceived health improvements from medication changes

Many participants felt it was beneficial to change their medication when their readings were too high, and were very pleased when they perceived that a medication change led to lower BP readings because of the positive effect this would have on their health.

"I've found that by having the medication changed up at regular intervals my blood pressure's improved all the time" (Intervention p15, well-controlled)

A few participants felt that a medication change had not been effective at lowering their BP which could create doubt about their medication's effectiveness.

"It's been doubled but it hasn't seemed to lower my blood pressure at all, in fact, it's at the same levels as it is sort of now, un-medicated. So I just think – I don't think it's the right one. You know, I can take the tablet but, actually, I don't think it's doing anything". (Intervention p26, poorly-controlled)

Intervention can facilitate management of side effects

Most participants did not experience any side effects from having their medication changed. Where side effects did occur, participants tended to perceive this as being a cost of taking medication

For peer review only - http://bmjopen.bmj.com/site/about/guidelines.xhtml

(which was balanced against the benefit of controlling BP), rather than a burden of the intervention itself. They felt that the intervention could help them to be more aware of side effects, to identify alternative medications and to monitor how these affect their health.

"That [side effect] would have happened, you know, no matter what. That would have been an issue but this has actually highlighted it, sort of, more clearly" (Intervention p5, poorly-controlled)

Burden of worrying about health

Negative emotional responses to seeing high readings

A burden of self-monitoring BP for some people was that seeing high readings could cause worry about health. Participants' beliefs about their BP control appeared to influence their appraisal of high readings. A few participants believed their BP was well-controlled, a belief which was perhaps reinforced by clinical staff approving their readings previously, and had only joined the study to help with research. These participants tended to feel shocked or annoyed when they received above-target feedback from the intervention as this challenged their beliefs.

"At one time, I was told to go on medication, further medication, which I must admit I was not very happy about… When I used to go for a check with the nurse, if I'd have had those particular readings, they wouldn't have been high" (Intervention p17, poorly controlled)

Others were confused or frustrated by high BP readings when they could not understand why this might have happened.

"I'm thinking about why my blood pressure has gone up. I can't think why" (Intervention p25, poorly controlled).

For peer review only - http://bmjopen.bmj.com/site/about/guidelines.xhtml

Meanwhile people who expected to see high readings were less concerned because they had accepted that high readings were likely.

“Just par for the course. It’s what I expect from my blood pressure, really, so, it never worries me” (Intervention p5, poorly controlled)

Perceptions about the causes of high BP also influenced how anxious people felt about seeing high readings. Those who felt that high readings held serious implications for their health tended to feel frightened. Some even felt apprehensive *before* self-monitoring in case their readings were out-of-range, as they didn't want to see evidence that their BP was too high or low.

“Before I take my blood pressure, I do get stressed. I wouldn't say I get massively stressed because obviously I'm used to doing it now but … it's just that apprehension and thinking 'Oh, God, I hope it's not too high today. I wonder really what's going on and how serious this is”. (Intervention p26, poorly controlled).

Other people were able to dismiss one-off high readings without feeling anxious as they attributed high readings to less threatening explanations such as feeling stressed, not sitting still for long enough, positioning of the cuff, or held a prior expectation of it being normal for BP to fluctuate. In these cases, the high readings had less negative emotional impact as they were not interpreted as indicating a serious underlying health issue.

Worrying about medication change affecting health

Some participants were worried about the effects that changing BP medication could have on their health. Previous experience of side effects, existence of co-morbidities, and concerns about medication dependency or impact on kidneys tended to make participants feel more worried about changing medication.

For peer review only

For peer review only - http://bmjopen.bmj.com/site/about/guidelines.xhtml

Perceptions about the health risk of high BP in terms of stroke and cardiovascular disease tended to affect how burdensome participants perceived a medication change to be. Anxiety about future health could override concerns about medication side effects or dependency as the behaviour was evaluated as beneficial in order to bring BP down, although sometimes participants still experienced conflict between the perceived benefit and burden.

"The blood pressure has gone down but now my worries have changed from blood pressure to other things. One is actually depending on medicine whole of my life. And secondly impact of medicine on my body like kidneys" (Intervention p16, well-controlled).

Burden of uncertainty from self-monitoring

Uncertainty about whether readings are representative

Whilst some participants were confident making decisions about when to monitor their BP, others were worried about whether their readings were representative, especially when BP was seen to vary at different times of day or after physical activity or drinking coffee. This could lead to doubt about the meaningfulness of self-monitoring and the recommendations of the intervention.

"I wonder if maybe the time of day I'm doing it, maybe my blood pressure's always gonna be roughly that. And could it be different during the day, is the sort of thing that does play in my mind a bit" (Intervention p1, well controlled).

Uncertainty about what to do about high or low readings

Uncertainty could also become a burden after seeing an out-of-range BP reading, as the participant had to decide what to do next. This burden was removed when the prescriber provided quick, personalised feedback to the participant, but when they did not receive any contact from their

For peer review only

prescriber after an out-of-range reading or felt the prescriber was not available to provide support, this could create a feeling of doubt.

"I suppose I knew there was nothing to worry about but it's always a bit of a niggle in the back of your mind… even the days she's [the nurse prescriber] at work I can't ring her at work because she may be, you know, doing something else" (Intervention p21, well-controlled)

Burden of thinking about making healthy lifestyle changes

Worry or guilt about not engaging with healthy changes

Several participants felt they would like to lose weight, eat more healthily, or do more physical activity but lacked the motivation or self-efficacy to make these changes, especially if they had other co-morbidities. This could create feelings of guilt or worry about their failure to make healthy changes, which was a burden of the intervention for them.

"I understand that, obviously, I need to get my blood pressure down because it is very dangerously high, but I just don't know what to do about it, you know?... where I feel fatigued and worn out, I don't feel well enough at the moment to do any exercise" (Intervention p26, poorly controlled)

Burden of the practicalities of adhering to intervention procedures

Burden of fitting self-monitoring into the day

Many participants felt that self-monitoring was easy to fit into their day, and some described this as being easier than going to the GP Surgery to have their BP taken. Those with busy daily lifestyles tended to find it harder to remember to self-monitor, and a burden for some participants was

For peer review only

For peer review only - http://bmjopen.bmj.com/site/about/guidelines.xhtml

deciding how best to fit self-monitoring into their routine given the instructions about not drinking coffee or exercising beforehand.

The perceived burden of regular self-monitoring seemed to be influenced by the perceived benefit of the behaviour, such that those who felt reassurance from seeing low readings or with high motivation to control BP found it less hassle and easier to remember than those who felt anxious about self-monitoring or had only joined the study to help with research.

"There was no big deal. It doesn’t take long and it’s—it’s quite nice to sit down and have a relax during the day" (Intervention p8, well-controlled)

For peer review only

For peer review only - http://bmjopen.bmj.com/site/about/guidelines.xhtml

DISCUSSION

This qualitative study has identified diverse perceived burdens and benefits of using a self-management digital intervention for high BP. In support of the BoT theory[1], the HOME BP intervention appeared to reduce the burden on patients to self-manage their condition by improving access to regular healthcare professional (HCP) support and facilitating better understanding of their condition, but in some cases there was a burden of worry about health or changing medication. How much benefit a patient perceived from the intervention compared to burden seemed to be influenced by the dynamics of the patient-HCP interaction (described as 'Improving Cooperation" in BoT theory) and the patient's own resources to manage their condition and cope with medication (described as "Capacity").

Another important factor relating to the burden experienced was personal beliefs about BP and treatment. Those who recognised that their BP was too high and did not have concerns about side effects or taking medication appeared to have more positive experiences of the intervention, perceiving self-monitoring as more worthwhile, and feeling less anxious about seeing high readings or changing medication. . This is consistent with the necessity-concerns framework[16]. BoT theory states that people who are better equipped with resources and are more resilient may cope better with the burden imposed by healthcare[17], but the importance of an individual's personal conceptualisation of their condition in how burdensome they find self-care is not strongly represented. This beliefs system may be partly encompassed by the "Relational Integration" aspect of BoT theory, which refers to the extent to which patients trust the tasks they do for healthcare, e.g. self-monitoring BP, and feel confident in the outcomes of these tasks, e.g. changing medication. However illness and treatment perceptions[18] are not explicitly covered by the theory and it may be helpful to consider them as additional factors which might influence the experience of treatment burden.

For peer review only - http://bmjopen.bmj.com/site/about/guidelines.xhtml

Implications for measurement of benefit and burden

The present study demonstrates the value of collecting in-depth qualitative data to develop a detailed understanding of the burden of treatment, and to discover perceptions specific to the context in which the intervention was implemented. The important psychosocial outcomes discovered using qualitative researchcan inform the selection or development of relevant quantitative measures to capture these factors in further evaluation.

Quantitative measures have been developed to appraise the structural aspects of burden of treatment[19 20], but these are not intended to assess psychosocial factors such as reassurance, anxiety or uncertainty which this study suggests can influence the extent to which this work is experienced subjectively as a burden.

Future research could explore how best to capture this. One approach might be to simply ask participants to quantify the net subjective burden or benefit of interventions. However, it could be challenging for participants to weigh complex heterogeneous psychosocial outcomes against one another and decide overall whether an intervention was more burdensome or beneficial. Capturing the extent to which patients experience positive or negative psychosocial outcomes might better assess how beneficial or burdensome the intervention was perceived to be. Although this would not produce a single outcome measure, cost-consequence analysis can be used to inform decision-making when an intervention has multiple relevant outcomes which cannot be aggregated into one value[21]. Coast[22] discusses whether a multidimensional approach is more informative for economic analysis or if a single aggregated value is more pragmatic.

Extending the evaluation of outcomes beyond health is in line with the capability approach[23], which focuses on broader aspects of subjective well-being which are not assessed by generic measures such as the EQ-5D[24]. Tools used to capture perceived capability (such as the ICECAP[25], and ASCOT[26])

For peer review only - http://bmjopen.bmj.com/site/about/guidelines.xhtml

are gaining support as holistic measures of economic evaluation, but do not assess the more specific psychosocial burdens and benefits of healthcare raised by participants in this study. Process utility emphasises the need to quantitatively measure the value that people attach to healthcare delivery. This approach might be relevant for evaluating how much value people perceive in the process of using digital health interventions and the capability this achieves[27]. It has been argued that process utility measures should also ask about the reasons behind patients' valuations, to better inform the decision-maker[28]. This would help to capture the individual differences found in this study in how people appraise the personal value of a digital intervention, informed by their underlying illness and treatment beliefs.

Strengths and limitations

A strength of the study was that we used relatively open questions formulated by a multi-disciplinary team which enabled us to elicit and explore a wide range of perceived burdens and benefits, some of which were not anticipated at the outset of research. We are aware of the lead researcher's potential influence on the data analysis, which we strived to minimise by transparent memoing of decisions and regular team meetings to discuss the emerging themes. Participants were sent newsletters to describe the findings of the study, but were not invited to provide feedback on the analysis.

We succeeded in speaking to well and poorly controlled hypertensive participants at different points in the intervention, although the uptake rate from those invited to interviews was not high, particularly in the usual care group. Perhaps unsurprisingly, it was difficult to recruit low engagers in the intervention group, which could have helped reach theoretical saturation. Despite this, there was a considerable range in the level of engagement of the intervention group patients in the study, and a wide range of patient demographics in terms of age, education level and gender.

For peer review only

Repeated interviews with the same participants may have offered more insight into the dynamic nature of perceived burdens and benefits over time, although more regular conversations about the target behaviour could have influenced participants' BP management behaviour therefore threatening the RCT conclusions. It has been noted that a key issue with process evaluations of interventions is the tendency for intervention content and impact to change over time[29], such that deciding the optimal point to collect evaluation data is challenging.

Some of the burdens and benefits described by patients in this study were also found to a lesser extent in the qualitative research undertaken during the development of the HOME BP intervention, such as reassurance from seeing well-controlled readings and knowing that the HCP was aware of your BP readings, as well as the concerns about side effects from medication changes and some evidence of worry about high readings or readings being unreliable due to variability[30]. Others were novel and only arose when participants experienced the full HOME BP intervention during the RCT as opposed to a prototype, for example the perceived health improvements from medication changes. This demonstrates the value of conducting inductive qualitative research to explore users' perspectives at each stage of intervention development and evaluation, in line with the person-based approach[8].

CONCLUSIONS

In the context of this digital intervention, the study shows that participants' appraisal of burdens and benefits appeared to be influenced by both intervention factors, such as BP readings and perceived availability of the healthcare professional, and patient characteristics, such as perceptions of BP control, previous experience of side effects, and co-morbidities. This nuanced evaluation would be lost in a population-level analysis, demonstrating the advantage of a more individualised approach for better understanding participants' perspectives of an intervention and how best to minimise the burden of treatment.

For peer review only - http://bmjopen.bmj.com/site/about/guidelines.xhtml

The study develops the recommendations of McNamee et al [6] that complex digital health interventions warrant a wider perspective for measuring health outcomes, and discusses the implications of capturing broader psychosocial outcomes for Burden of Treatment theory and health economic evaluations.

The finding that some participants perceived personal benefits from using the intervention demonstrates that the process of healthcare can, in itself, be positive for some people, highlighting the importance of capturing transient short-term benefits to take these into account as well as the burden of self-management.

For peer review only

For peer review only - http://bmjopen.bmj.com/site/about/guidelines.xhtml

CONTRIBUTORS

KM recruited participants, conducted interviews, analysed data, and wrote manuscript. LD & KB contributed to study design and data analysis. KB and RB developed online intervention. PL & RJM contributed to intervention development and interpretation of themes. CM & JR contributed to theoretical and methodological implications of the study findings. LY contributed to study design, data collection, data analysis, and interpretation.

All authors contributed to the manuscript preparation and provided final approval of the version to be published.

-

- **FUNDING**

This independent research was funded by the National Institute for Health Research (NIHR) Programme Grants for Applied Research Programme (Grant Reference Number RP-PG-1211-20001). The views expressed are those of the author(s) and not necessarily those of the NHS, the NIHR, or the Department of Health. RJM is funded by an NIHR Professorship (NIHR-RP-R2-12-015) and the NIHR Oxford CLAHRC.

- **COMPETING INTERESTS**

RJM has received BP monitors for research purposes from Omron and Lloyds Pharmacies.

- **ETHICS APPROVAL**

- This qualitative process study was embedded in the Home BP trial and approved by the University of Southampton and NHS Hampshire A Research Ethics committees (REC Reference 15/SC/0082).

DATA SHARING STATEMENT

For peer review only - http://bmjopen.bmj.com/site/about/guidelines.xhtml

Requests for data sharing can be sent to the corresponding author. Full transcripts of interviews are not available to protect participants' anonymity.

For peer review only

For peer review only - http://bmjopen.bmj.com/site/about/guidelines.xhtml

REFERENCES

1. May CR, Eton DT, Boehmer K, et al. Rethinking the patient: using Burden of Treatment Theory to understand the changing dynamics of illness. *BMC Health Serv Res* 2014;**14**(1):281.

2. Eton DT, de Oliveira DR, Egginton JS, et al. Building a measurement framework of burden of treatment in complex patients with chronic conditions: a qualitative study. *Patient related outcome measures* 2012;**3**:39.

3. Gallacher K, May CR, Montori VM, et al. Understanding patients' experiences of treatment burden in chronic heart failure using normalization process theory. *The Annals of Family Medicine* 2011;**9**(3):235-43.

4. Sanders GD, Neumann PJ, Basu A, et al. Recommendations for conduct, methodological practices, and reporting of cost-effectiveness analyses: second panel on cost-effectiveness in health and medicine. *JAMA* 2016;**316**(10):1093-103.

5. Ioannidis JP, Garber AM. Individualized cost-effectiveness analysis. *PLoS Med* 2011;**8**(7):e1001058.

6. McNamee P, Murray E, Kelly MP, et al. Designing and undertaking a health economics study of digital health interventions. *Am J Prev Med* 2016;**51**(5):852-60.

7. Band R, Morton K, Stuart B, et al. Home and Online Management and Evaluation of Blood Pressure (HOME BP) digital intervention for self-management of uncontrolled, essential hypertension: a protocol for the randomised controlled HOME BP trial. *BMJ open* 2016;**6**(11):e012684.

8. Yardley L, Morrison L, Bradbury K, et al. The person-based approach to intervention development: application to digital health-related behavior change interventions. *J Med Internet Res* 2015;**17**(1).

9. Tong A, Sainsbury P, Craig J. Consolidated criteria for reporting qualitative research (COREQ): a 32-item checklist for interviews and focus groups. *Int J Qual Health Care* 2007;**19**(6):349-57.

10. Braun V, Clarke V. Using thematic analysis in psychology. *Qualitative research in psychology* 2006;**3**(2):77-101.

11. Joffe H, Yardley L. Content and thematic analysis. *Research methods for clinical and health psychology* 2004;**56**:68.

For peer review only

For peer review only - http://bmjopen.bmj.com/site/about/guidelines.xhtml

12. Strauss A, Corbin JM. *Grounded theory in practice*: Sage, 1997.

13. Charmaz K. *Constructing grounded theory*: Sage, 2014.

14. NVivo qualitative data analysis Software [program], 2012.

15. Rogers PJ, Stevens K, Boymal J. Qualitative cost-benefit evaluation of complex, emergent programs. *Eval Program Plann* 2009;**32**(1):83-90.

16. Horne R, Weinman J. Patients' beliefs about prescribed medicines and their role in adherence to treatment in chronic physical illness. *J Psychosom Res* 1999;**47**(6):555-67.

17. Mair FS, May CR. Thinking about the burden of treatment. *Br Med J* 2014;**349**:g6680.

18. Leventhal H, Diefenbach M, Leventhal EA. Illness cognition: using common sense to understand treatment adherence and affect cognition interactions. *Cognit Ther Res* 1992;**16**(2):143-63.

19. Eton DT, Yost KJ, Lai J-s, et al. Development and validation of the Patient Experience with Treatment and Self-management (PETS): a patient-reported measure of treatment burden. *Quality of life research: an international journal of quality of life aspects of treatment, care and rehabilitation* 2017;**26**(2):489-503.

20. Tran V-T, Harrington M, Montori VM, et al. Adaptation and validation of the Treatment Burden Questionnaire (TBQ) in English using an internet platform. *BMC Med* 2014;**12**(1):109.

21. NICE. Incorporating economic evaluation. 2014. https://www.nice.org.uk/process/pmg20/chapter/incorporating-economic-evaluation.12th July 2017

22. Coast J, Kinghorn P, Mitchell P. The development of capability measures in health economics: opportunities, challenges and progress. *The Patient-Patient-Centered Outcomes Research* 2015;**8**(2):119-26.

23. Nussbaum M, Sen A. *The quality of life*: Clarendon Press - Oxford, 1993.

24. Brazier J, Ratcliffe J, Saloman J, et al. *Measuring and valuing health benefits for economic evaluation*: OXFORD university press, 2017.

25. Al-Janabi H, Flynn TN, Coast J. Development of a self-report measure of capability wellbeing for adults: the ICECAP-A. *Qual Life Res* 2012;**21**(1):167-76.

For peer review only

26. Netten A, Burge P, Malley J, et al. Outcomes of social care for adults: developing a preference-weighted measure. *Health Technol Assess* 2012;**16**(16).

27. Ryan M, Kinghorn P, Entwistle VA, et al. Valuing patients' experiences of healthcare processes: Towards broader applications of existing methods. *Soc Sci Med* 2014;**106**:194-203.

28. Donaldson C, Shackley P. Does "process utility" exist? A case study of willingness to pay for laparoscopic cholecystectomy. *Soc Sci Med* 1997;**44**(5):699-707.

29. Moore GF, Audrey S, Barker M, et al. Process evaluation of complex interventions: Medical Research Council guidance. *BMJ* 2015;**350**:h1258.

30. Bradbury K, Morton K, Grist R, et al. Using the Person-Based Approach to optimise a digital intervention for the management of hypertension. *In submission* 2017.

For peer review only

For peer review only - http://bmjopen.bmj.com/site/about/guidelines.xhtml

For peer review only

For peer review only - http://bmjopen.bmj.com/site/about/guidelines.xhtml

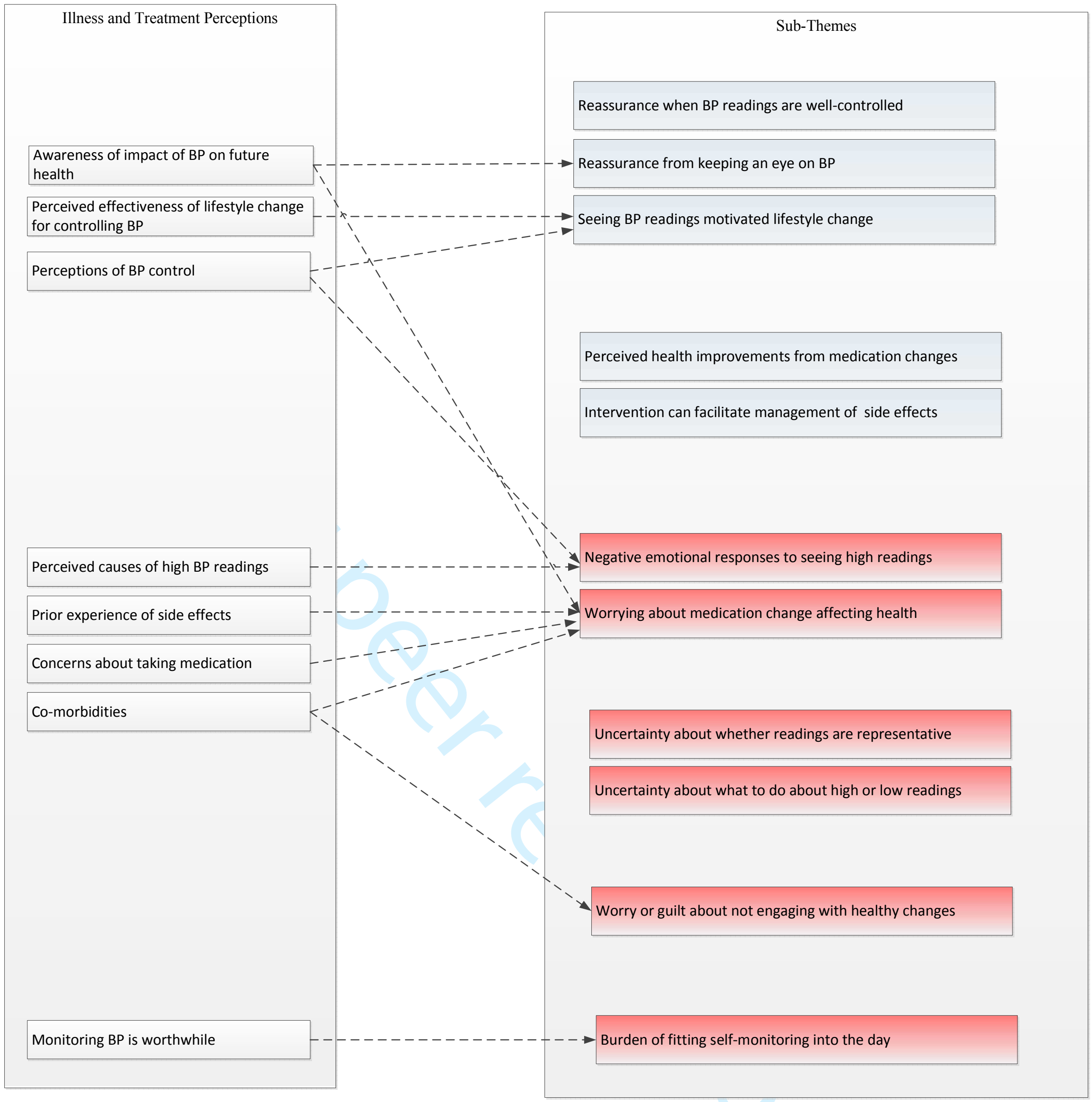

Figure 1 Possible influences of illness and treatment beliefs on perceived burdens and benefits of the intervention

peer review only

Additional File 1. Interview schedules for intervention and usual care group

Intervention group interview schedule

Opening questions

Can you tell me about what it is like for you to have high blood pressure?

How did you feel when you were first told you had high blood pressure?

Can you tell me why you decided to sign up for the Home BP study?

I'm really interested in hearing about your experiences of using HOME BP, can you tell me all about it?

Can you tell me about anything that you have liked about the HOME BP study so far?

Can you tell me about anything that you have disliked?

Can you tell me about any advantages of using HOME BP?

And can you tell me about any disadvantages of using HOME BP?

How do you feel about your high blood pressure now?

Do you think you would choose to keep on using the HOME BP programme if it was still available after a year?

Why/ Why not?

Would you recommend the HOME BP programme to other people with high blood pressure?

Self-monitoring

For peer review only

Experiences of monitoring:

Can you tell me about what it was like when you were learning how to use your BP monitor at home?

Can you tell me about the first time you used your BP monitor at home in the study?

Can you tell me about what it has been like to monitor your blood pressure yourself?

How did it make you feel?

Had you ever monitored your own blood pressure before you took part in the HOME BP study?

If yes:

Can you tell me a bit more about this?

Can you tell me whether you've noticed any changes in how you monitor your blood pressure since you've started the HOME BP programme?

All: Can you tell me about any concerns that you have about monitoring your blood pressure?

Adherence to the monitoring schedule:

The HOME BP programme reminds you to monitor your blood pressure for 7 days every 4 weeks.

Can you tell me about what it has been like for you to try and monitor your blood pressure for 7 days every 4 weeks?

Can you tell me about the time of day you have been monitoring your blood pressure so far?

How do you feel about the instructions HOME BP gives about when to monitor?

Was there anything that helped you to monitor your own blood pressure?

For peer review only

Was there anything that made it harder to monitor your own blood pressure?

Can you tell me about any time when you had to skip your blood pressure monitoring or change when you did it in the day?

Can you tell me how you found it entering your readings on the HOME BP programme?

Feedback messages

Remember after you enter your readings on HOME BP, you see a message which says 'your readings were amber, or green'? What did you think about these messages?

Can you tell me about how you felt when you saw a message about your blood pressure readings on HOME BP?

How did you feel about monitoring your own blood pressure after reading that message?

Did you have any very high or very low readings (red/blue) when you were monitoring in the HOME BP study? How did you feel?

2. Medication change

Can you tell me about what it's like for you taking medication for your high blood pressure?

Have you been recommended to make a medication change during the HOME BP study?

If yes:

How did you feel about making a change to your medication when HOME BP recommended it?

Did you make this change to your medication?

If they chose to make a change:

For peer review only

Can you tell me about what it was like to make a medication change in the HOME BP study?

Can you tell me about how the medication change came about?

How did you get on with your new medication?

How would you feel if HOME BP recommended a medication change for you now?

If they chose not to make a change –

Can you tell me about why you decided not to change your medication?

How would you feel if HOME BP recommended another medication change for you now?

If no:

If patient has ***not*** *been recommended a med change:* How do you feel about changing your medication if your blood pressure stayed too high for too long?

Can you tell me about any concerns that you have about doing this?

All:

How did you feel about making changes to your medication before you took part in HOME BP?

3. HCP support

Can you tell me about the contact you have had with your GP or nurse so far in the HOME BP study?

Can you tell me about how you have found the support from your GP or nurse while you were taking part in the HOME BP study?

Can you tell me what you thought about receiving emails from your nurse about HOME BP?

If needed, follow-up questions:

Have you seen your GP about your blood pressure since the start of the study?

For peer review only

And what about appointments with the nurse?

Can you tell me about how you have found the emails from HOME BP?

4. Healthy changes

Can you tell me about anything else that you do to try and manage your high blood pressure?

There is an option on the HOME BP programme to look at online sessions about making healthy changes that can help lower your blood pressure, I wonder if you remember if you have seen it?

If no:

Can you tell about how you might feel about making healthy changes to help lower your blood pressure?

If yes:

Can you tell me about how you found the session on healthy changes?

How did you feel about making the healthy changes this session talked about?

If they chose a change: Can you tell me about what made you choose this/these ones?

How did you find the online session about xxxx (cutting down on salt/alcohol/eating a healthier diet/doing more physical activity/ losing weight)?

Can you tell me about what it was like for you trying out this healthy change?

How did making a healthy change like this make you feel?

Or if they didn't choose any: Can you tell me about why you didn't want to choose a healthy change?

All: Can you tell me about any concerns that you have about making a new healthy change?

For peer review only

Is there anything else you would like to tell me about the HOME BP study that we haven't already talked about?

Usual care group interview schedule

General questions about BP

Can you tell me about what it is like for you to have high blood pressure?

How did you feel when you were first told you had high blood pressure?

Can you talk me through what (if anything) you currently do to manage/control your high blood pressure?

Can you tell me about how your GP and practice nurse are involved in helping you to control your high blood pressure?

How do you feel about monitoring your own blood pressure at home?

If they already monitor – can you tell me a bit more about this?

Can you tell me about what it's like for you taking medication for your high blood pressure?

Can you tell me how you feel about your GP making changes to your blood pressure medication?

HOME BP study experience

Can you tell me why you signed up for the Home BP study?

For peer review only

Can you tell me all about what it has been like for you being in the HOME BP study in the usual care group?

At the start of the HOME BP study, you had a baseline appointment with a nurse at your GP Practice – can you tell me about that?

How did you find it using the HOME BP programme to fill in your questionnaires online at the start of the study?

After you had completed your questionnaires, did you have an appointment with your GP to check your blood pressure medication? Can you tell me about this?

HCP support

Can you tell me about the contact you have had with your GP or nurse so far in the HOME BP study?

Can you tell me about how you have found the support from your GP or nurse while you were taking part in the HOME BP study?

For peer review only

Consolidated criteria for reporting qualitative studies (COREQ): 32-item checklist

Developed from:
Tong A, Sainsbury P, Craig J. Consolidated criteria for reporting qualitative research (COREQ): a 32-item checklist for interviews and focus groups. *International Journal for Quality in Health Care*. 2007. Volume 19, Number 6: pp. 349 – 357

YOU MUST PROVIDE A RESPONSE FOR ALL ITEMS. ENTER N/A IF NOT APPLICABLE

No. Item	Guide questions/description	Reported on Page #
**Domain 1: Research team and reflexivity**		
Personal Characteristics		
1. Inter viewer/facilitator	Which author/s conducted the inter view or focus group?	Page 9
2. Credentials	What were the researcher's credentials? E.g. PhD, MD	Page 9
3. Occupation	What was their occupation at the time of the study?	Page 9
4. Gender	Was the researcher male or female?	Page 9
5. Experience and training	What experience or training did the researcher have?	Page 9
Relationship with participants		
6. Relationship established	Was a relationship established prior to study commencement?	N/A
7. Participant knowledge of the interviewer	What did the participants know about the researcher? e.g. personal goals, reasons for doing the research	N/A
8. Interviewer characteristics	What characteristics were reported about the inter viewer/facilitator? e.g. Bias, assumptions, reasons and interests in the research topic	Page 9
**Domain 2: study design**		
Theoretical framework		
9. Methodological orientation and Theory	What methodological orientation was stated to underpin the study? e.g. grounded theory, discourse analysis, ethnography, phenomenology, content analysis	Page 9
Participant selection		
10. Sampling	How were participants selected? e.g. purposive, convenience, consecutive, snowball	Page 8
11. Method of approach	How were participants approached? e.g. face-to-face, telephone, mail, email	Page 8

For peer review only

12. Sample size	How many participants were in the study?	Page 10
13. Non-participation	How many people refused to participate or dropped out? Reasons?	Page 10
Setting		
14. Setting of data collection	Where was the data collected? e.g. home, clinic, workplace	Page 8
15. Presence of non-participants	Was anyone else present besides the participants and researchers?	N/A
16. Description of sample	What are the important characteristics of the sample? e.g. demographic data, date	Page 10
Data collection		
17. Interview guide	Were questions, prompts, guides provided by the authors? Was it pilot tested?	Page 8
18. Repeat interviews	Were repeat inter views carried out? If yes, how many?	N/A
19. Audio/visual recording	Did the research use audio or visual recording to collect the data?	Page 9
20. Field notes	Were field notes made during and/or after the inter view or focus group?	Page 9
21. Duration	What was the duration of the inter views or focus group?	Page 10
22. Data saturation	Was data saturation discussed?	Page 8
23. Transcripts returned	Were transcripts returned to participants for comment and/or correction?	N/A
**Domain 3: analysis and findings**		
Data analysis		
24. Number of data coders	How many data coders coded the data?	Page 9
25. Description of the coding tree	Did authors provide a description of the coding tree?	Page 12
26. Derivation of themes	Were themes identified in advance or derived from the data?	Page 9
27. Software	What software, if applicable, was used to manage the data?	Page 9
28. Participant checking	Did participants provide feedback on the findings?	Page 25
Reporting		
29. Quotations presented	Were participant quotations presented to illustrate the themes/findings? Was each quotation identified? e.g. participant number	Throughout results
30. Data and findings consistent	Was there consistency between the data presented and the findings?	Yes
31. Clarity of major themes	Were major themes clearly presented in the findings?	Yes
32. Clarity of minor themes	Is there a description of diverse cases or discussion of minor themes?	Yes

For peer review only

Once you have completed this checklist, please save a copy and upload it as part of your submission. When requested to do so as part of the upload process, please select the file type: *Checklist*. You will NOT be able to proceed with submission unless the checklist has been uploaded. Please DO NOT include this checklist as part of the main manuscript document. It must be uploaded as a separate file.

For peer review only

BMJ Open

A qualitative process study to explore the perceived burdens and benefits of a digital intervention for self-managing high blood pressure in Primary Care.

Journal:	*BMJ Open*
Manuscript ID	bmjopen-2017-020843.R1
Article Type:	Research
Date Submitted by the Author:	01-Feb-2018
Complete List of Authors:	Morton, Katherine; University of Southampton, Psychology<br>Dennison, Laura; University of Southampton, Psychology<br>Bradbury, Katherine; University of Southampton, Psychology<br>Band, Rebecca; University of Southampton, Psychology<br>May, Carl; University of Southampton, Faculty of Health Sciences<br>Raftery, James; Southampton University, School of Medicine<br>Little, Paul; University of Southampton, Primary Care and Population Science<br>McManus, Richard; University of Oxford, Dept of Primary Care Health Sciences<br>Yardley, Lucy ; University of Oxford, Nuffield Department of Primary Health Care Sciences
**Primary Subject Heading**:	Qualitative research
Secondary Subject Heading:	Cardiovascular medicine, Health services research, Public health
Keywords:	Self-management, digital intervention, qualitative, treatment burden

SCHOLARONE™ Manuscripts

Title page

A qualitative process study to explore the perceived burdens and benefits of a digital intervention for self-managing high blood pressure in Primary Care.

Kate Morton[a]*, Laura Dennison[a], Katherine Bradbury[a], Rebecca Band[a], Carl May[b], James Raftery[c], Paul Little[d], Richard J. McManus[e], & Lucy Yardley[a]

a. Academic unit of psychology, University of Southampton, Southampton, UK
b. Faculty of Health Sciences, University of Southampton, Southampton, UK
c. Faculty of Medicine, University of Southampton, Southampton, UK
d. Primary Care Research, University of Southampton, Southampton, UK
e. Nuffield Department of Primary Care Health Sciences, University of Oxford, Oxford, UK

*Corresponding author: ksm1r13@soton.ac.uk; 02380 592234

Room 3063, B44, University of Southampton, Southampton, SO17 1BJ

Word count 5047

For peer review only

For peer review only - http://bmjopen.bmj.com/site/about/guidelines.xhtml

ABSTRACT

Objectives: Digital interventions can change patients' experiences of managing their health, either creating additional burden or improving their experience of healthcare. This qualitative study aimed to explore perceived burdens and benefits for patients using a digital self-management intervention for reducing high blood pressure. A secondary aim was to further our understanding of how best to capture burdens and benefits when evaluating health interventions.

Design: Inductive qualitative process study nested in a randomised controlled trial.

Setting: Primary Care in the UK

Participants: 35 participants taking antihypertensive medication and with uncontrolled blood pressure at baseline participated in semi-structured telephone interviews.

Intervention: Digital self-management intervention to support blood pressure self-monitoring and medication change when recommended by the healthcare professional.

Analysis: Data were analysed using inductive thematic analysis with techniques from grounded theory.

Results: Seven themes were developed which reflected perceived burdens and benefits of using the intervention, including worry about health, uncertainty about self-monitoring, and reassurance. The analysis showed how beliefs about their condition and treatment appeared to influence participants' appraisal of the value of the intervention. This suggested that considering illness and treatment perceptions in Burden of Treatment theory could further our understanding of how individuals appraise the personal costs and benefits of self-managing their health.

For peer review only

Conclusions: Patients' appraisal of the burden or benefit of using a complex self-management intervention seemed to be influenced by experiences within the intervention (such as perceived availability of support) and beliefs about their condition and treatment (such as perceived control and risk of side effects). Developing our ability to adequately capture these salient burdens and benefits for patients could help enhance evaluation of self-management interventions in the future. Many participants perceived important benefits from using the intervention, highlighting the need for theory to recognise that engaging in self-management can include positive as well as negative aspects.

Trial registration: ISRCTN13790648. Registered 14 May 2015.

Keywords: Self-management; digital intervention; qualitative; treatment burden

Strengths and limitations of this study:

- The exploratory, open approach to data collection enabled us to capture whichever benefits or burdens were most salient to the participants.
- We only interviewed participants at one point in time, so were unable to gain an understanding of dynamic changes in perceived benefits or burdens over time.
- Both well and poorly controlled hypertensive patients took part in the interviews, but it was difficult to recruit low users of the intervention which could limit the generalisability of the findings.
- The asymptomatic nature of hypertension and the unique medication change pathway means that these findings may not be generalisable across conditions.

For peer review only

BACKGROUND

The work involved in looking after one's health when living with a chronic condition can include complex tasks such as organising and adhering to treatment regimes, interacting with healthcare professionals (HCPs), regular monitoring of health indicators, and making health-related decisions, all of which can accumulate into a considerable burden [1]. Digital self-management interventions are often developed to improve health outcomes, but these interventions could also either increase or minimise the burden of the healthcare process for patients. Developing our understanding of the burdens of self-management can help to better optimise the delivery of healthcare to improve adherence and well-being[1-3]. Burden of Treatment (BoT) theory provides a mechanism for understanding these experiences in the context of patients' personal capacity to cope, with emphasis on the role of wider healthcare systems and social networks available to the patient[1].

Health economic evaluations also focus on understanding the impact of healthcare on patients, seeking to weigh up the resources used against the health outcomes in order to better inform decision-making. Recent guidelines for economic evaluations in health and medicine recommend adopting a societal perspective such that all relevant outcomes are evaluated, rather than focusing only on formal healthcare costs[4]. In particular, personal costs such as time spent in self-care should be included. Consequently, BoT theory and health economic evaluations share an interest in adequately capturing the wider burdens or personal costs of engaging with healthcare. For consistency in terminology in this paper, negative outcomes/personal costs of healthcare will be referred to as 'burdens'.

BoT theory considers patients' time as a resource that is used by the healthcare system, while health economic evaluation counts time as an 'opportunity cost' whereby the patient 'spends' time that could have been spent on something other than healthcare. However, subjective experiences of time spent on digital interventions may be varied and complex. Heterogeneity in the relative value placed on the outcomes of the intervention[5] may mean that for some participants the time spent

For peer review only - http://bmjopen.bmj.com/site/about/guidelines.xhtml

engaging with elements of an intervention is not perceived as a burden but rather as a benefit, either because it is interesting, pleasant or meaningful in and of itself or because of the positive outcomes it can lead to. In other words, some people may actually like engaging with healthcare. The value of exploring the personal benefits of intervention participation has not received as much focus as understanding the costs, such as treatment burden. McNamee et al.[6] proposed that the health research guidelines for economic analysis may need to be adjusted for digital health interventions to ensure we can fully capture the heterogeneous costs and benefits arising when complex interventions are implemented in complex systems.

To further our understanding of how patients perceive benefits and burdens when using digital health interventions, we carried out a qualitative process study [7]. The digital HOME BP intervention was developed based on best practice recommendations to help improve hypertension in poorly controlled patients by facilitating self-monitoring of blood pressure (BP) at home and prompting appropriate intensification of medication by HCPs[8]. This intervention could help to minimise the treatment burden of hypertension by providing an online healthcare system in which HCPs have sight of patients' home readings, streamlining the process for finding the most effective medication without the need for attending the GP surgery. However, HOME BP is a complex, interactive multi-component intervention, which creates potential diversity in the perceived burden and benefits for participants using it. The contexts in which the intervention is embedded may also be diverse, and factors such as individual differences in patients' health status, beliefs about medication and risks of high BP, availability of time and resources, and access to support may influence how the intervention is perceived and valued. The HOME BP intervention was developed using the person-based approach[9] which emphasises the importance of understanding participants' unique perspectives and different situations when developing and implementing digital interventions. Adopting a more granular approach to the evaluation of benefit and burden is consistent with the

For peer review only

For peer review only - http://bmjopen.bmj.com/site/about/guidelines.xhtml

person-based approach, and with the BoT approach of fully understanding the participants' perspective.

The present study aimed to explore the perceived burden and benefits of using a digital health intervention for self-managing BP using qualitative process interviews with intervention and usual care participants taking part in a randomised controlled trial (RCT).This paper seeks to interpret the implications for optimising the capture of perceived costs and benefits in health economic evaluations and evaluating the burden of treatment.

METHODS

Design

A qualitative process study embedded in the HOME BP trial[8] was approved by the University of Southampton and NHS Research Ethics committees. The COREQ checklist (Consolidated criteria for reporting qualitative studies) was used to ensure comprehensive reporting of the study[10] (supplementary file 1).

Intervention

The HOME BP programme supports participants to self-manage their high BP, primarily via home self-monitoring of BP and making changes to dose/drug type when recommended by the HCP. Lifestyle change modules are also available, but optional as the key target behaviours for the intervention were self-monitoring and medication change adherence [8 11]. Participants using HOME BP were supported by a 'prescriber' (GP or nurse prescriber responsible for changing medication)

For peer review only - http://bmjopen.bmj.com/site/about/guidelines.xhtml

and a ‘supporter’ (nurse or healthcare assistant who supported participants in self-monitoring and choosing lifestyle changes).

Participants were invited to use the online programme by their GP and were randomised to usual care or intervention after completing baseline measures online. Those randomised to the intervention group completed two online training sessions which sought to overcome concerns about variability in readings and changing medication. Participants were encouraged to monitor their BP in the mornings, but the programme allowed flexibility as it was most important that people found a time of day that suited them to monitor their BP. Both intervention and usual care participants were followed-up at 6 and 12 months post-randomisation.

Table 1 describes the HOME BP intervention in more detail.

Table 1 HOME BP Intervention Characteristics

Target behaviour	Description
Self-monitoring BP	Participants monitored their BP at home for 7 days every 4 weeks. After 7 days, they entered their BP readings on the HOME BP website and received instant automated feedback using a traffic light system. If BP was very high (red) or very low (blue), they were told to contact their GP surgery. If BP was above target (amber), they were told their prescriber would contact them about a medication change. If BP was on target (green), they were congratulated and asked to monitor their BP again next time.
Medication change	The prescriber planned three potential medication changes with the participant at the start of the study. HOME BP informed prescribers by email when a patient’s home BP readings were above-target and they could implement a pre-

For peer review only

For peer review only - http://bmjopen.bmj.com/site/about/guidelines.xhtml

	planned change without needing to see the participant for an appointment.
Optional lifestyle changes	At nine weeks after randomisation, participants had the option of choosing an online session to support lifestyle change to help control their BP, specifically weight management, salt reduction, healthy diet, physical activity, or alcohol reduction. Participants were alerted by email when this became available, and saw an option to view the healthy lifestyles session each time they logged on to HOME BP. The online lifestyle change sessions could be started at any time during the 12-month trial, from nine weeks.

For peer review only

Participants

Patients were eligible to take part in the HOME BP trial if they had uncontrolled hypertension (mean BP reading of 140/90 mm Hg or more at baseline taken at the GP Surgery using a validated electronic automated sphygmomanometer (BP TRU BPM 200)) managed in Primary Care, were prescribed 1-3 antihypertensive medications at baseline, and aged over 18 (full inclusion and exclusion criteria are listed in the protocol[8]).

Both intervention and usual care participants were invited to take part in interviews as we felt that obtaining an understanding of managing BP in usual care would aid interpretation of the perceived burden and benefits of the intervention. We aimed to speak to participants at a range of time-points during the 12-month trial from 10 weeks onwards as this gave participants the opportunity to become familiar with HOME BP. No new intervention content was introduced after the lifestyles sessions became available at nine weeks.

For peer review only - http://bmjopen.bmj.com/site/about/guidelines.xhtml

Recruitment and interview procedure

A sub-sample of RCT participants were invited by email to provide feedback on their experiences of managing their BP (n=78, of 622 patients in the RCT). Informed consent was taken by post or online, depending on participant preference. Recruitment was initially opportunistic, but subsequently a purposive approach was adopted to target younger participants, low engagers, and those with recent uncontrolled self-monitored BP readings, informed by the concurrent analysis. Recruitment was stopped once the researchers agreed that data saturation had been reached and no new burdens or benefits were arising.

Semi-structured interview schedules were co-developed by experts in health psychology (KM, KB, RB, LY, LD), health economics (JR) and sociology (CM). Open, inductive questions were carefully selected to elicit data about the burden and benefits of BP management perceived as most salient by the participants (see Supplementary File 2 for interview schedules). The interviews were conducted by telephone to minimise the burden on participants, except in one case where the participant asked to meet face-to-face due to struggling with hearing on the telephone. The interviews took place between February 2016 and February 2017. Each participant was given a £10 gift voucher to thank them for their time.

All interviews were conducted by KM (MSc, BSc. termed “the researcher”), a female PhD candidate in Health Psychology who was also employed as a research assistant. Each interview was audio-recorded, and the researcher also took notes and completed a self-reflection log afterwards to record any emerging thoughts on the data. Audio-recordings were transcribed verbatim and checked thoroughly by the researcher.

Analysis

The analysis was an iterative process led by KM, supported by frequent discussion of emerging themes with LY and LD (who have extensive experience in qualitative research) along with input

For peer review only

For peer review only - http://bmjopen.bmj.com/site/about/guidelines.xhtml

regarding health economic and sociological perspectives (JR and CM). Inductive thematic analysis methods were used[12 13] with techniques from grounded theory such as memoing, constant comparison, and diagramming to enhance our understanding and facilitate the development of higher themes[14 15]. Data collection and analysis ran concurrently to enable purposive sampling based on analytic insights. Thorough line-by-line coding was undertaken in NVivo 10[16], and a coding manual was developed which evolved as more data were collected and coded. The emerging codes were constantly checked against the raw data to ensure the analysis was driven by the participants' own language and experiences.

All data relating to burdens and benefits of managing BP were analysed. We also coded factors that appeared to influence perceptions of burdens and benefits to facilitate an in-depth understanding of how participants appraised the intervention's value. A broad and open definition was adopted whereby benefits and burdens were defined as positive and negative outcomes or experiences of engaging in the intervention[17], in order to facilitate a comprehensive representation of all potentially relevant data.

RESULTS

Participant characteristics

In the intervention group, 28 of 54 invited participants agreed to be interviewed (52%). In the usual care group, 7 of 24 invited participants agreed (29%). Most participants who did not take part chose not to reply, but those who did said they did not have anything to report on the trial (n = 3 in usual care). The participants were from 19 different GP surgeries. Table 2 shows the sociodemographic and intervention details of the sample.

For peer review only - http://bmjopen.bmj.com/site/about/guidelines.xhtml

Table 2. Sociodemographic and intervention participant data (n=35)

	Intervention participants	Usual care participants
N	28	7
Average duration of interview (range)	39 (15-67) minutes	28 (22-40) minutes
Average age (range)	65 (41-87) years	67 (52-77) years
Gender	71% female	43% female
Ethnicity<br><br>White<br>Black African<br>Pakistani<br>Other	 <br>24<br>1<br>1<br>2	 <br>6<br><br><br>1
Education levels	9 No formal education	2 No formal education
	8 GCSE or A-level	3 GCSE or A-level
	10 Higher Education	1 Higher Education
	1 Other	1 Other
Number of weeks since randomisation	23 (10-57) weeks	17 (7 to 24) weeks
Poorly controlled BP at the time of the interview	10/28 (36%)	N/A*
Medication change recommended during the study	15/28 (54%)	N/A
Accessed optional healthy lifestyles session	15/28 (54%)	N/A

For peer review only

For peer review only - http://bmjopen.bmj.com/site/about/guidelines.xhtml

*As BP self-monitoring was a key component of the intervention, BP readings were available for the intervention group throughout the duration of the study but data about BP from the usual care group were only available at RCT baseline and follow-up points.

Themes

Table 3 presents seven themes exploring perceived burdens and benefits of the HOME BP intervention. One meta-theme also emerged concerning how illness and treatment beliefs about high BP appeared to influence participants' perceptions about the intervention's burdens and benefits, and this is discussed in relation to each theme it applies to. Figure 1 shows how illness and treatment perceptions about BP appeared to relate to the sub-themes identified by the thematic analysis.

Where quotes are included, participants are referred to as 'p' followed by a number. Study group (intervention or usual care) is also included to help understand the quotes in context.

For peer review only

For peer review only - http://bmjopen.bmj.com/site/about/guidelines.xhtml

Table 3. Themes and sub-themes relating to perceived burdens and benefits of the intervention

Themes	Sub-themes	Exemplar participant quote
Benefit of reassurance from seeing BP readings	Reassurance when BP readings are well-controlled	"I'm so pleased. And my mind is at rest when we go on holidays and all that...I'm alright. I'm alright sort of thing. Yeah, peace of mind" (Intervention p9, well-controlled)
	Reassurance from keeping an eye on BP	"It made me much more aware of what the problem is with the high blood pressure and by monitoring it so regularly, I know exactly where I stand with it" (Intervention p15, well-controlled)
Benefit of motivation for lifestyle change from seeing BP readings	Seeing BP readings motivated lifestyle change	"It is quite interesting to see the effects of what I'm doing on the blood pressure and everything. So, I think that is – it is quite good" (Intervention p18, well-controlled)
Benefit of better	Perceived health improvements	"It helped me to change my medication and then because of change of medication,

For peer review only

health	from medication changes	my blood pressure went down. So definitely there is a benefit" (Intervention p16, well-controlled)
	Intervention can facilitate management of side effects	"That medication didn't work, in that I was on holiday and my ankles swelled up so much – and my feet and my legs, so much so that I couldn't see my toes. So I stopped taking that medication. Was called back to the GP. And I'm now on a medication that works for me and is managing the blood pressure" (Intervention p7, well-controlled)
Burden of worrying about health	Negative emotional responses to seeing high readings	"I was actually quite shocked because it was a—a lot higher" (Intervention p6, poorly controlled)
	Worrying about medication change affecting health	"I don't want to get more medication 'cause I'm already on a high dose and I don't want to increase it because it worries me about my kidneys" (Intervention p24, poorly controlled)
Burden of uncertainty from self-monitoring	Uncertainty about whether readings are representative	"If someone only ever takes it in the morning, and you tend to get those lower readings, are you really getting a true picture of what they're like in the afternoon or the evening?" (Intervention p10, well-controlled)

For peer review only - http://bmjopen.bmj.com/site/about/guidelines.xhtml

	Uncertainty about what to do about high or low readings	"I don't know what's going to happen in respect to that [amber feedback]. Whether I'm going to get a call from my GP, or whether he – so I'm a little bit, like, you know, in the air. I don't really know what's going to happen in that respect" (Intervention p22, poorly controlled)
Burden of thinking about making healthy lifestyle changes	Worry or guilt about not engaging with healthy changes	"I have looked at it [online healthy lifestyles session]. I wouldn't say I've looked at it seriously, and I need to" (Intervention p4, poorly controlled)
Burden of the practicalities of adhering to intervention procedures	Burden of fitting self-monitoring into the day	"I like to get up and have a cup of coffee and I'm thinking 'Well, let's get the blood pressure done first because otherwise I can't do that, you know, for a while afterwards.' So, I've found that quite—quite difficult" (Intervention p5, poorly controlled).

219

For peer review only - http://bmjopen.bmj.com/site/about/guidelines.xhtml

For peer review only

For peer review only - http://bmjopen.bmj.com/site/about/guidelines.xhtml

Insert Figure 1 here

Benefit of reassurance from seeing BP readings

Reassurance when BP readings are well-controlled

Seeing well-controlled readings when self-monitoring BP gave participants peace of mind which was widely perceived as a benefit of the intervention. People described feeling relieved that their BP readings were lower than at the GP surgery, and felt this gave them more insight into what their BP was like most of the time.

"What I do like about it is taking the blood pressure here at home, the readings are lower. And I find that quite reassuring that my blood pressure is not always high" (Intervention p11, well-controlled)

Several usual care participants had decided to use their own BP monitors, and this group also described feeling reassurance when seeing their BP was well-controlled.

Reassurance from keeping an eye on BP

Most participants liked having an increased focus on their BP through regular monitoring and found it interesting to compare their readings over time. However one participant perceived that taking BP regularly could encourage too much attention on your health, which was a potential burden of the intervention for her (Intervention p28, BP control unknown as did not enter BP readings on HOME BP). This participant had low concern about her BP generally, and was not motivated to engage in self-management.

Even when participants had poorly-controlled readings, many felt a benefit from the intervention as it enabled them to regularly check their BP and detect any problems instantly rather than carrying on unaware.

For peer review only

"I think it's helping me to know where my blood pressure stands because it's a regular thing every month" (Intervention p24, poorly-controlled)

The knowledge that home readings were shared with the prescriber reassured participants as they knew that any problems would not only be detected but also dealt with at the time, making them feel well cared for. This contrasted with the perceived burden of managing BP in usual care where some participants felt concerned that their GP did not change their medication when their home readings were too high, or would have liked more regular contact with their Practice to check their BP and medication.

"It would be nice to have it checked, I guess, you know, every three months or whatever. How—however often. I mean, how do they know that everything is working?" (Usual care p4).

This shows that although participants in usual care gained reassurance from seeing low readings when they monitored at home, the lack of interaction with the Practice could cause concern when readings were high or when patients did not regularly monitor BP at home of their own accord.

Benefit of motivation for lifestyle change from seeing BP readings

Some participants were motivated to increase their physical activity, engage in stress management activities or healthy eating because they could see this had a positive impact on their BP readings. This helped them feel more in control of their BP.

"By taking the readings regularly and frequently, it gave me more of a feedback straightaway if you like about anything, changes that I did make like a bit of exercise or…practicing relaxation and this sort of thing. So that was quite nice, it was nice to feel that I was more in control of it again" (Intervention p20, well-controlled)

For peer review only

For peer review only - http://bmjopen.bmj.com/site/about/guidelines.xhtml

Other participants felt frustrated after making lifestyle changes in the past which had no effect on their BP. This made them feel that lifestyle was ineffective for controlling BP.

“I’m a completely different person. My diet’s completely different. And my blood pressure remained the same. So I’ve done literally everything you physically possibly can to help yourself, and nothing’s worked” (Intervention p1, well-controlled).

Benefit of better health

Perceived health improvements from medication changes

Many participants felt it was beneficial to change their medication when their readings were too high, and were very pleased when they perceived that a medication change led to lower BP readings because of the positive effect this would have on their health.

“I’ve found that by having the medication changed up at regular intervals my blood pressure’s improved all the time” (Intervention p15, well-controlled)

A few participants felt that a medication change had not been effective at lowering their BP which could create doubt about their medication’s effectiveness.

“It's been doubled but it hasn't seemed to lower my blood pressure at all, in fact, it's at the same levels as it is sort of now, un-medicated. So I just think – I don't think it's the right one. You know, I can take the tablet but, actually, I don't think it's doing anything”. (Intervention p26, poorly-controlled)

Intervention can facilitate management of side effects

Most participants did not experience any side effects from having their medication changed. Where side effects did occur, participants tended to perceive this as being a cost of taking medication

For peer review only

For peer review only - http://bmjopen.bmj.com/site/about/guidelines.xhtml

(which was balanced against the benefit of controlling BP), rather than a burden of the intervention itself. They felt that the intervention could help them to be more aware of side effects, to identify alternative medications and to monitor how these affect their health.

"That [side effect] would have happened, you know, no matter what. That would have been an issue but this has actually highlighted it, sort of, more clearly" (Intervention p5, poorly-controlled)

Burden of worrying about health

Negative emotional responses to seeing high readings

A burden of self-monitoring BP for some people was that seeing high readings could cause worry about health. Participants' beliefs about their BP control appeared to influence their appraisal of high readings. A few participants believed their BP was well-controlled, a belief which was perhaps reinforced by clinical staff approving their readings previously, and had only joined the study to help with research. These participants tended to feel shocked or annoyed when they received above-target feedback from the intervention as this challenged their beliefs.

"At one time, I was told to go on medication, further medication, which I must admit I was not very happy about… When I used to go for a check with the nurse, if I'd have had those particular readings, they wouldn't have been high" (Intervention p17, poorly controlled)

Others were confused or frustrated by high BP readings when they could not understand why this might have happened.

"I'm thinking about why my blood pressure has gone up. I can't think why" (Intervention p25, poorly controlled).

For peer review only - http://bmjopen.bmj.com/site/about/guidelines.xhtml

Meanwhile people who expected to see high readings were less concerned because they had accepted that high readings were likely.

“Just par for the course. It’s what I expect from my blood pressure, really, so, it never worries me” (Intervention p5, poorly controlled)

Perceptions about the causes of high BP also influenced how anxious people felt about seeing high readings. Those who felt that high readings held serious implications for their health tended to feel frightened. Some even felt apprehensive *before* self-monitoring in case their readings were out-of-range, as they didn't want to see evidence that their BP was too high or low.

“Before I take my blood pressure, I do get stressed. I wouldn't say I get massively stressed because obviously I'm used to doing it now but … it's just that apprehension and thinking 'Oh, God, I hope it's not too high today. I wonder really what's going on and how serious this is”. (Intervention p26, poorly controlled).

Other people were able to dismiss one-off high readings without feeling anxious as they attributed high readings to less threatening explanations such as feeling stressed, not sitting still for long enough, positioning of the cuff, or held a prior expectation of it being normal for BP to fluctuate. In these cases, the high readings had less negative emotional impact as they were not interpreted as indicating a serious underlying health issue.

Worrying about medication change affecting health

Some participants were worried about the effects that changing BP medication could have on their health. Previous experience of side effects, existence of co-morbidities, and concerns about medication dependency or impact on kidneys tended to make participants feel more worried about changing medication.

For peer review only

For peer review only - http://bmjopen.bmj.com/site/about/guidelines.xhtml

Perceptions about the health risk of high BP in terms of stroke and cardiovascular disease tended to affect how burdensome participants perceived a medication change to be. Anxiety about future health could override concerns about medication side effects or dependency as the behaviour was evaluated as beneficial in order to bring BP down, although sometimes participants still experienced conflict between the perceived benefit and burden.

“The blood pressure has gone down but now my worries have changed from blood pressure to other things. One is actually depending on medicine whole of my life. And secondly impact of medicine on my body like kidneys” (Intervention p16, well-controlled).

For peer review only

Burden of uncertainty from self-monitoring

Uncertainty about whether readings are representative

Whilst some participants were confident making decisions about when to monitor their BP, others were worried about whether their readings were representative, especially when BP was seen to vary at different times of day or after physical activity or drinking coffee. This could lead to doubt about the meaningfulness of self-monitoring and the recommendations of the intervention.

“I wonder if maybe the time of day I’m doing it, maybe my blood pressure’s always gonna be roughly that. And could it be different during the day, is the sort of thing that does play in my mind a bit” (Intervention p1, well controlled).

Uncertainty about what to do about high or low readings

Uncertainty could also become a burden after seeing an out-of-range BP reading, as the participant had to decide what to do next. This burden was removed when the prescriber provided quick, personalised feedback to the participant, but when they did not receive any contact from their

For peer review only - http://bmjopen.bmj.com/site/about/guidelines.xhtml

prescriber or felt the prescriber was not available to provide support, this could create a feeling of doubt.

"I suppose I knew there was nothing to worry about but it's always a bit of a niggle in the back of your mind… even the days she's [the nurse prescriber] at work I can't ring her at work because she may be, you know, doing something else" (Intervention p21, well-controlled)

Burden of thinking about making healthy lifestyle changes

Worry or guilt about not engaging with healthy changes

Several participants felt they would like to lose weight, eat more healthily, or do more physical activity but lacked the motivation or self-efficacy to make these changes, especially if they had other co-morbidities. This could create feelings of guilt or worry about their failure to make healthy changes, which was a burden of the intervention for them.

"I understand that, obviously, I need to get my blood pressure down because it is very dangerously high, but I just don't know what to do about it, you know?... where I feel fatigued and worn out, I don't feel well enough at the moment to do any exercise" (Intervention p26, poorly controlled)

Burden of the practicalities of adhering to intervention procedures

Burden of fitting self-monitoring into the day

Many participants felt that self-monitoring was easy to fit into their day, and some described this as being easier than going to the GP Surgery to have their BP taken. Those with busy daily lifestyles tended to find it harder to remember to self-monitor, and a burden for some participants was

For peer review only

For peer review only - http://bmjopen.bmj.com/site/about/guidelines.xhtml

deciding how best to fit self-monitoring into their routine given the instructions about not drinking coffee or exercising beforehand.

The perceived burden of regular self-monitoring seemed to be mitigated by the perceived benefit of the behaviour, such that those who felt reassurance from seeing low readings or with high motivation to control BP found it less hassle and easier to remember than those who felt anxious about self-monitoring or had only joined the study to help with research.

"There was no big deal. It doesn’t take long and it’s—it’s quite nice to sit down and have a relax during the day" (Intervention p8, well-controlled)

For peer review only

For peer review only - http://bmjopen.bmj.com/site/about/guidelines.xhtml

DISCUSSION

This qualitative study has identified diverse perceived burdens and benefits of using a self-management digital intervention for high BP. In support of the BoT theory[1], the HOME BP intervention appeared to reduce the burden on patients to self-manage their condition by improving access to regular HCP support and facilitating better understanding of their condition, but in some cases there was a burden of worry about health or changing medication. How much benefit a patient perceived from the intervention compared to burden seemed to be influenced by the dynamics of the patient-HCP interaction (described as 'Improving Cooperation" in BoT theory) and the patient's own resources to manage their condition and cope with medication (described as "Capacity").

Another important factor relating to the burden experienced was personal beliefs about BP and treatment. Those who recognised that their BP was too high and did not have concerns about side effects or taking medication appeared to have more positive experiences of the intervention, perceiving self-monitoring as more worthwhile, and feeling less anxious about seeing high readings or changing medication. This is consistent with the necessity-concerns framework[18]. BoT theory states that people who are better equipped with resources and are more resilient may cope better with the burden imposed by healthcare[19], but the importance of an individual's personal conceptualisation of their condition in how burdensome they find self-care is not strongly represented. This beliefs system may be partly encompassed by the "Relational Integration" aspect of BoT theory, which refers to the extent to which patients trust the tasks they do for healthcare, e.g. self-monitoring BP, and feel confident in the outcomes of these tasks, e.g. changing medication. However illness and treatment perceptions[20] are not explicitly covered by the theory and it may be helpful to consider them as additional factors which might influence the experience of treatment burden.

For peer review only - http://bmjopen.bmj.com/site/about/guidelines.xhtml

406

407 **Implications for measurement of benefit and burden**

408 The present study demonstrates the value of collecting in-depth qualitative data to develop a
409 detailed understanding of the burden of treatment, and to discover perceptions specific to the
410 context in which the intervention was implemented. The important psychosocial outcomes
411 discovered using qualitative research can inform the selection or development of relevant
412 quantitative measures to capture these factors in further evaluation.

413 Quantitative measures have been developed to appraise the structural aspects of burden of
414 treatment[21 22], but these are not intended to assess psychosocial factors such as reassurance, anxiety
415 or uncertainty which this study suggests can influence the extent to which using an intervention is
416 experienced subjectively as a burden.

417 Future research could explore how best to capture the perceived burden or benefit of an
418 intervention. One approach might be to simply ask participants to quantify the net subjective burden
419 or benefit of interventions. However, it could be challenging for participants to weigh complex
420 heterogeneous psychosocial outcomes against one another and decide overall whether an
421 intervention was more burdensome or beneficial. Capturing the extent to which patients experience
422 positive or negative psychosocial outcomes might better assess how beneficial or burdensome the
423 intervention was perceived to be. Although this would not produce a single outcome measure, cost-
424 consequence analysis can be used to inform decision-making when an intervention has multiple
425 relevant outcomes which cannot be aggregated into one value[23]. Coast[24] discusses whether a
426 multidimensional approach is more informative for economic analysis or if a single aggregated value
427 is more pragmatic.

428 Extending the evaluation of outcomes beyond health is in line with the capability approach[25], which
429 focuses on broader aspects of subjective well-being which are not assessed by generic measures

For peer review only - http://bmjopen.bmj.com/site/about/guidelines.xhtml

such as the EQ-5D[26]. Tools used to capture perceived capability (such as the ICECAP[27], and ASCOT[28]) are gaining support as holistic measures of economic evaluation, but do not assess the more specific psychosocial burdens and benefits of healthcare raised by participants in this study. Process utility emphasises the need to quantitatively measure the value that people attach to healthcare delivery. This approach might be relevant for evaluating how much value people perceive in the process of using digital health interventions and the capability this achieves[29]. It has been argued that process utility measures should also ask about the reasons behind patients' valuations, to better inform the decision-maker[30]. This would help to capture the individual differences found in this study in how people appraise the personal value of a digital intervention, informed by their underlying illness and treatment beliefs.

Strengths and limitations

A strength of the study was that we used relatively open questions formulated by a multi-disciplinary team which enabled us to elicit and explore a wide range of perceived burdens and benefits, some of which were not anticipated at the outset of research. We are aware of the lead researcher's potential influence on the data analysis, which we strived to minimise by transparent memoing of decisions and regular team meetings to discuss the emerging themes. Participants were sent newsletters to describe the findings of the study, but were not invited to provide feedback on the analysis.

We succeeded in speaking to well and poorly controlled hypertensive participants at different points in the intervention, and there was a wide range of demographics in terms of age, education level and gender in the sample. However, the uptake rate from those invited to interviews was not high, particularly in the usual care group. Perhaps unsurprisingly, it was difficult to recruit low engagers in the intervention group, which could have helped reach theoretical saturation. In terms of wider applicability, we are aware that these findings may not be generalizable across other health conditions, as the lack of symptoms in hypertension and the stepped pathway for changing medication are quite unique features of this condition.

For peer review only - http://bmjopen.bmj.com/site/about/guidelines.xhtml

For peer review only

Repeated interviews with the same participants may have offered more insight into the dynamic nature of perceived burdens and benefits over time, although more regular conversations about the target behaviour could have influenced participants' BP management behaviour therefore threatening the RCT conclusions. It has been noted that a key issue with process evaluations of interventions is the tendency for intervention content and impact to change over time[7], such that deciding the optimal point to collect evaluation data is challenging.

Some of the burdens and benefits described by patients in this study were also found to a lesser extent in the qualitative development of the HOME BP intervention, such as reassurance from seeing well-controlled readings, and some concerns about side effects and high or variable readings [31]. Others were novel and only arose when participants experienced the full HOME BP intervention during the RCT as opposed to a prototype, for example the perceived health improvements from medication changes. This demonstrates the value of conducting inductive qualitative research to explore users' perspectives at each stage of intervention development and evaluation, in line with the person-based approach[9].

CONCLUSIONS

In the context of this digital intervention, the study shows that participants' appraisal of burdens and benefits appeared to be influenced by both intervention factors, such as BP readings and perceived availability of the healthcare professional, and patient characteristics, such as perceptions of BP control, previous experience of side effects, and co-morbidities. This nuanced evaluation would be lost in a population-level analysis, demonstrating the advantage of a more individualised approach for better understanding participants' perspectives of an intervention and how best to minimise the burden of treatment.

For peer review only

The study develops the recommendations of McNamee et al [6] that complex digital health interventions warrant a wider perspective for measuring health outcomes, and discusses the implications of capturing broader psychosocial outcomes for Burden of Treatment theory and health economic evaluations.

The finding that some participants perceived personal benefits from using the intervention demonstrates that the process of healthcare can, in itself, be positive for some people, highlighting the importance of capturing transient short-term benefits to take these into account as well as the burden of self-management.

For peer review only

For peer review only - http://bmjopen.bmj.com/site/about/guidelines.xhtml

CONTRIBUTORS

KM recruited participants, conducted interviews, analysed data, and wrote manuscript. LD & KB contributed to study design and data analysis. KB and RB developed the digital intervention. PL & RJM contributed to intervention development and interpretation of themes. CM & JR contributed to theoretical and methodological implications of the study findings. LY contributed to study design, data collection, data analysis, and interpretation.

All authors contributed to the manuscript preparation and provided final approval of the version to be published.

FUNDING

This independent research was funded by the National Institute for Health Research (NIHR) Programme Grants for Applied Research Programme (Grant Reference Number RP-PG-1211-20001). The views expressed are those of the author(s) and not necessarily those of the NHS, the NIHR, or the Department of Health. RJM is funded by an NIHR Professorship (NIHR-RP-R2-12-015) and the NIHR Oxford CLAHRC.

COMPETING INTERESTS

RJM has received BP monitors for research purposes from Omron and Lloyds Pharmacies.

ETHICS APPROVAL

This qualitative process study was embedded in the HOME BP trial and approved by the University of Southampton and NHS Hampshire A Research Ethics committees (REC Reference 15/SC/0082).

DATA SHARING STATEMENT

For peer review only - http://bmjopen.bmj.com/site/about/guidelines.xhtml

Requests for data sharing can be sent to the corresponding author. Full transcripts of interviews are not available to protect participants' anonymity.

Figure legend:

Figure 1 Possible influences of illness and treatment beliefs on perceived burdens and benefits of the intervention

For peer review only

REFERENCES

1. May CR, Eton DT, Boehmer K, et al. Rethinking the patient: using Burden of Treatment Theory to understand the changing dynamics of illness. *BMC Health Serv Res* 2014;**14**(1):281.

2. Eton DT, de Oliveira DR, Egginton JS, et al. Building a measurement framework of burden of treatment in complex patients with chronic conditions: a qualitative study. *Patient Relat Outcome Meas* 2012;**3**:39.

3. Gallacher K, May CR, Montori VM, et al. Understanding patients' experiences of treatment burden in chronic heart failure using normalization process theory. *Ann Fam Med* 2011;**9**(3):235-43.

4. Sanders GD, Neumann PJ, Basu A, et al. Recommendations for conduct, methodological practices, and reporting of cost-effectiveness analyses: second panel on cost-effectiveness in health and medicine. *JAMA* 2016;**316**(10):1093-103.

5. Ioannidis JP, Garber AM. Individualized cost-effectiveness analysis. *PLoS Med* 2011;**8**(7):e1001058.

6. McNamee P, Murray E, Kelly MP, et al. Designing and undertaking a health economics study of digital health interventions. *Am J Prev Med* 2016;**51**(5):852-60.

7. Moore GF, Audrey S, Barker M, et al. Process evaluation of complex interventions: Medical Research Council guidance. *BMJ* 2015;**350**:h1258.

8. Band R, Morton K, Stuart B, et al. Home and Online Management and Evaluation of Blood Pressure (HOME BP) digital intervention for self-management of uncontrolled, essential hypertension: a protocol for the randomised controlled HOME BP trial. *BMJ open* 2016;**6**(11):e012684.

9. Yardley L, Morrison L, Bradbury K, et al. The person-based approach to intervention development: application to digital health-related behavior change interventions. *J Med Internet Res* 2015;**17**(1).

10. Tong A, Sainsbury P, Craig J. Consolidated criteria for reporting qualitative research (COREQ): a 32-item checklist for interviews and focus groups. *Int J Qual Health Care* 2007;**19**(6):349-57.

For peer review only

For peer review only - http://bmjopen.bmj.com/site/about/guidelines.xhtml

11. Band R, Bradbury K, Morton K, et al. Intervention planning for a digital intervention for self-management of hypertension: a theory-, evidence-and person-based approach. *Implementation Science* 2017;**12**(1):25.

12. Braun V, Clarke V. Using thematic analysis in psychology. *Qual Res Psychol* 2006;**3**(2):77-101.

13. Marks DF, Yardley L. *Research methods for clinical and health psychology*: Sage, 2004.

14. Strauss A, Corbin JM. *Grounded theory in practice*: Sage, 1997.

15. Charmaz K. *Constructing grounded theory*: Sage, 2014.

16. NVivo qualitative data analysis Software [program], 2012.

17. Rogers PJ, Stevens K, Boymal J. Qualitative cost-benefit evaluation of complex, emergent programs. *Eval Program Plann* 2009;**32**(1):83-90.

18. Horne R, Weinman J. Patients' beliefs about prescribed medicines and their role in adherence to treatment in chronic physical illness. *J Psychosom Res* 1999;**47**(6):555-67.

19. Mair FS, May CR. Thinking about the burden of treatment. *Br Med J* 2014;**349**:g6680.

20. Leventhal H, Diefenbach M, Leventhal EA. Illness cognition: using common sense to understand treatment adherence and affect cognition interactions. *Cognit Ther Res* 1992;**16**(2):143-63.

21. Eton DT, Yost KJ, Lai J-s, et al. Development and validation of the Patient Experience with Treatment and Self-management (PETS): a patient-reported measure of treatment burden. *Qual Life Res* 2017;**26**(2):489-503.

22. Tran V-T, Harrington M, Montori VM, et al. Adaptation and validation of the Treatment Burden Questionnaire (TBQ) in English using an internet platform. *BMC Med* 2014;**12**(1):109.

23. NICE. Incorporating economic evaluation. 2014. https://www.nice.org.uk/process/pmg20/chapter/incorporating-economic-evaluation.12th July 2017

24. Coast J, Kinghorn P, Mitchell P. The development of capability measures in health economics: opportunities, challenges and progress. *Patient* 2015;**8**(2):119-26.

For peer review only - http://bmjopen.bmj.com/site/about/guidelines.xhtml

25. Nussbaum M, Sen A. *The quality of life*: Clarendon Press - Oxford, 1993.

26. Brazier J, Ratcliffe J, Saloman J, et al. *Measuring and valuing health benefits for economic evaluation*: OXFORD university press, 2017.

27. Al-Janabi H, Flynn TN, Coast J. Development of a self-report measure of capability wellbeing for adults: the ICECAP-A. *Qual Life Res* 2012;**21**(1):167-76.

28. Netten A, Burge P, Malley J, et al. Outcomes of social care for adults: developing a preference-weighted measure. *Health Technol Assess* 2012;**16**(16).

29. Ryan M, Kinghorn P, Entwistle VA, et al. Valuing patients' experiences of healthcare processes: Towards broader applications of existing methods. *Soc Sci Med* 2014;**106**:194-203.

30. Donaldson C, Shackley P. Does “process utility” exist? A case study of willingness to pay for laparoscopic cholecystectomy. *Soc Sci Med* 1997;**44**(5):699-707.

31. Bradbury K, Morton K, Grist R, et al. Using the Person-Based Approach to optimise a digital intervention for the management of hypertension. *In submission* 2017.

For peer review only

For peer review only - http://bmjopen.bmj.com/site/about/guidelines.xhtml

For peer review only

For peer review only - http://bmjopen.bmj.com/site/about/guidelines.xhtml

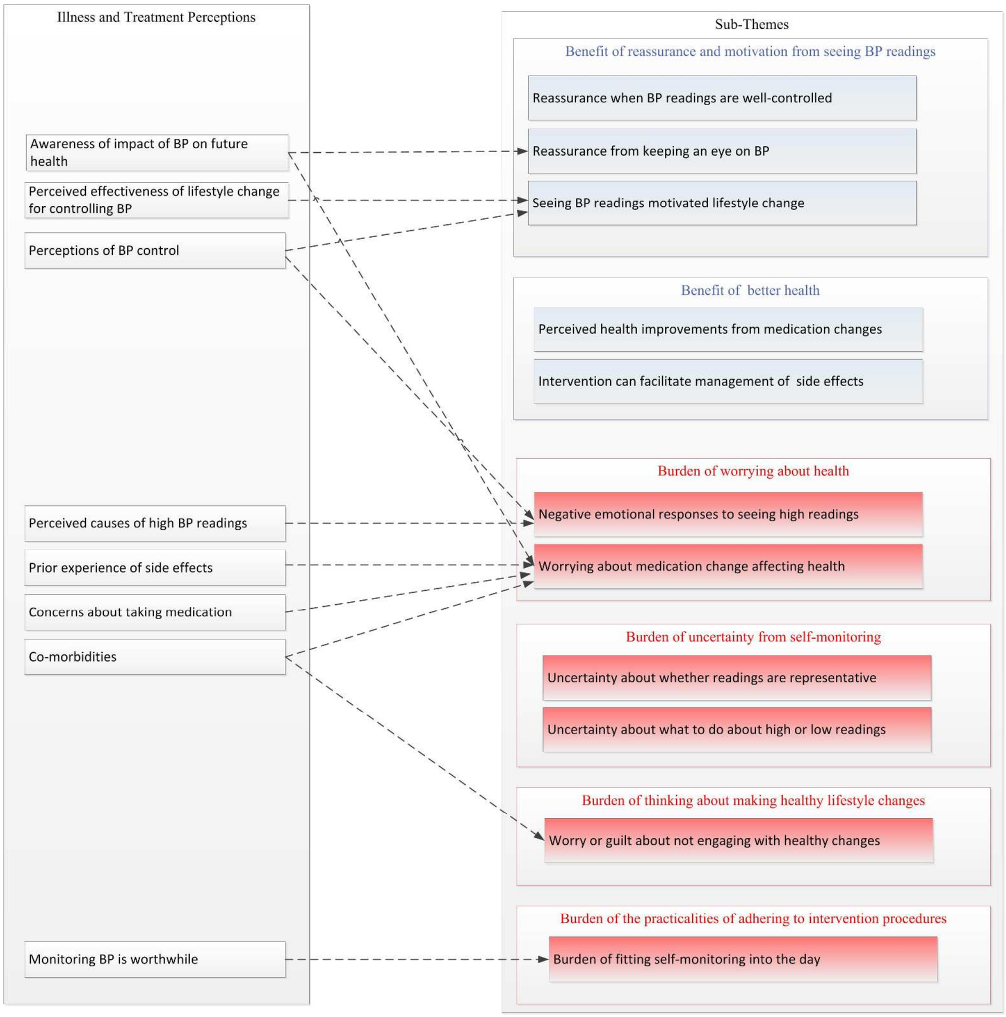

Figure 1. Possible influences of illness and treatment beliefs on perceived burdens and benefits of the intervention

337x340mm (300 x 300 DPI)

Consolidated criteria for reporting qualitative studies (COREQ): 32-item checklist

Developed from:
Tong A, Sainsbury P, Craig J. Consolidated criteria for reporting qualitative research (COREQ): a 32-item checklist for interviews and focus groups. *International Journal for Quality in Health Care*. 2007. Volume 19, Number 6: pp. 349 – 357

YOU MUST PROVIDE A RESPONSE FOR ALL ITEMS. ENTER N/A IF NOT APPLICABLE

No. Item	Guide questions/description	Reported on Page #
**Domain 1: Research team and reflexivity**		
Personal Characteristics		
1. Interviewer/facilitator	Which author/s conducted the interview or focus group?	Page 9
2. Credentials	What were the researcher's credentials? E.g. PhD, MD	Page 9
3. Occupation	What was their occupation at the time of the study?	Page 9
4. Gender	Was the researcher male or female?	Page 9
5. Experience and training	What experience or training did the researcher have?	Page 9
Relationship with participants		
6. Relationship established	Was a relationship established prior to study commencement?	N/A
7. Participant knowledge of the interviewer	What did the participants know about the researcher? e.g. personal goals, reasons for doing the research	N/A
8. Interviewer characteristics	What characteristics were reported about the interviewer/facilitator? e.g. Bias, assumptions, reasons and interests in the research topic	Page 9
**Domain 2: study design**		
Theoretical framework		
9. Methodological orientation and Theory	What methodological orientation was stated to underpin the study? e.g. grounded theory, discourse analysis, ethnography, phenomenology, content analysis	Page 9
Participant selection		
10. Sampling	How were participants selected? e.g. purposive, convenience, consecutive, snowball	Page 8
11. Method of approach	How were participants approached? e.g. face-to-face, telephone, mail, email	Page 8

12. Sample size	How many participants were in the study?	Page 10
13. Non-participation	How many people refused to participate or dropped out? Reasons?	Page 10
Setting		
14. Setting of data collection	Where was the data collected? e.g. home, clinic, workplace	Page 8
15. Presence of non-participants	Was anyone else present besides the participants and researchers?	N/A
16. Description of sample	What are the important characteristics of the sample? e.g. demographic data, date	Page 11
Data collection		
17. Interview guide	Were questions, prompts, guides provided by the authors? Was it pilot tested?	Page 9
18. Repeat interviews	Were repeat inter views carried out? If yes, how many?	N/A
19. Audio/visual recording	Did the research use audio or visual recording to collect the data?	Page 9
20. Field notes	Were field notes made during and/or after the interview or focus group?	Page 9
21. Duration	What was the duration of the inter views or focus group?	Page 11
22. Data saturation	Was data saturation discussed?	Page 9
23. Transcripts returned	Were transcripts returned to participants for comment and/or correction?	N/A
**Domain 3: analysis and findings**		
Data analysis		
24. Number of data coders	How many data coders coded the data?	Page 9
25. Description of the coding tree	Did authors provide a description of the coding tree?	Page 13
26. Derivation of themes	Were themes identified in advance or derived from the data?	Page 9-10
27. Software	What software, if applicable, was used to manage the data?	Page 10
28. Participant checking	Did participants provide feedback on the findings?	Page 26
Reporting		
29. Quotations presented	Were participant quotations presented to illustrate the themes/findings? Was each quotation identified? e.g. participant number	Throughout results
30. Data and findings consistent	Was there consistency between the data presented and the findings?	Yes
31. Clarity of major themes	Were major themes clearly presented in the findings?	Yes
32. Clarity of minor themes	Is there a description of diverse cases or discussion of minor themes?	Yes

For peer review only

Once you have completed this checklist, please save a copy and upload it as part of your submission. When requested to do so as part of the upload process, please select the file type: *Checklist*. You will NOT be able to proceed with submission unless the checklist has been uploaded. Please DO NOT include this checklist as part of the main manuscript document. It must be uploaded as a separate file.

For peer review only

Supplementary File 2. Interview schedules for intervention and usual care group

Intervention group interview schedule

Opening questions

Can you tell me about what it is like for you to have high blood pressure?

How did you feel when you were first told you had high blood pressure?

Can you tell me why you decided to sign up for the Home BP study?

I'm really interested in hearing about your experiences of using HOME BP, can you tell me all about it?

Can you tell me about anything that you have liked about the HOME BP study so far?

Can you tell me about anything that you have disliked?

Can you tell me about any advantages of using HOME BP?

And can you tell me about any disadvantages of using HOME BP?

How do you feel about your high blood pressure now?

Do you think you would choose to keep on using the HOME BP programme if it was still available after a year?

Why/ Why not?

Would you recommend the HOME BP programme to other people with high blood pressure?

Self-monitoring

Experiences of monitoring:

Can you tell me about what it was like when you were learning how to use your BP monitor at home?

Can you tell me about the first time you used your BP monitor at home in the study?

For peer review only

Can you tell me about what it has been like to monitor your blood pressure yourself?

How did it make you feel?

Had you ever monitored your own blood pressure before you took part in the HOME BP study?

If yes:

Can you tell me a bit more about this?

Can you tell me whether you've noticed any changes in how you monitor your blood pressure since you've started the HOME BP programme?

All: Can you tell me about any concerns that you have about monitoring your blood pressure?

Adherence to the monitoring schedule:

The HOME BP programme reminds you to monitor your blood pressure for 7 days every 4 weeks.

Can you tell me about what it has been like for you to try and monitor your blood pressure for 7 days every 4 weeks?

Can you tell me about the time of day you have been monitoring your blood pressure so far?

How do you feel about the instructions HOME BP gives about when to monitor?

Was there anything that helped you to monitor your own blood pressure?

Was there anything that made it harder to monitor your own blood pressure?

Can you tell me about any time when you had to skip your blood pressure monitoring or change when you did it in the day?

Can you tell me how you found it entering your readings on the HOME BP programme?

For peer review only

Feedback messages

Remember after you enter your readings on HOME BP, you see a message which says ‘your readings were amber, or green’? What did you think about these messages?

Can you tell me about how you felt when you saw a message about your blood pressure readings on HOME BP?

How did you feel about monitoring your own blood pressure after reading that message?

Did you have any very high or very low readings (red/blue) when you were monitoring in the HOME BP study? How did you feel?

2. Medication change

Can you tell me about what it’s like for you taking medication for your high blood pressure?

Have you been recommended to make a medication change during the HOME BP study?

If yes:

How did you feel about making a change to your medication when HOME BP recommended it?

Did you make this change to your medication?

If they chose to make a change:

Can you tell me about what it was like to make a medication change in the HOME BP study?

Can you tell me about how the medication change came about?

How did you get on with your new medication?

How would you feel if HOME BP recommended a medication change for you now?

For peer review only

If they chose not to make a change –

Can you tell me about why you decided not to change your medication?

How would you feel if HOME BP recommended another medication change for you now?

If no:

If patient has ***not*** *been recommended a med change:* How do you feel about changing your medication if your blood pressure stayed too high for too long?

Can you tell me about any concerns that you have about doing this?

All:

How did you feel about making changes to your medication before you took part in HOME BP?

3. HCP support

Can you tell me about the contact you have had with your GP or nurse so far in the HOME BP study?

Can you tell me about how you have found the support from your GP or nurse while you were taking part in the HOME BP study?

Can you tell me what you thought about receiving emails from your nurse about HOME BP?

If needed, follow-up questions:

Have you seen your GP about your blood pressure since the start of the study?

And what about appointments with the nurse?

Can you tell me about how you have found the emails from HOME BP?

4. Healthy changes

Can you tell me about anything else that you do to try and manage your high blood pressure?

For peer review only

There is an option on the HOME BP programme to look at online sessions about making healthy changes that can help lower your blood pressure, I wonder if you remember if you have seen it?

If no:

Can you tell about how you might feel about making healthy changes to help lower your blood pressure?

If yes:

Can you tell me about how you found the session on healthy changes?

How did you feel about making the healthy changes this session talked about?

If they chose a change: Can you tell me about what made you choose this/these ones?

How did you find the online session about xxxx (cutting down on salt/alcohol/eating a healthier diet/doing more physical activity/ losing weight)?

Can you tell me about what it was like for you trying out this healthy change?

How did making a healthy change like this make you feel?

Or if they didn't choose any: Can you tell me about why you didn't want to choose a healthy change?

All: Can you tell me about any concerns that you have about making a new healthy change?

Is there anything else you would like to tell me about the HOME BP study that we haven't already talked about?

For peer review only

Usual care group interview schedule

General questions about BP

Can you tell me about what it is like for you to have high blood pressure?

How did you feel when you were first told you had high blood pressure?

Can you talk me through what (if anything) you currently do to manage/control your high blood pressure?

Can you tell me about how your GP and practice nurse are involved in helping you to control your high blood pressure?

How do you feel about monitoring your own blood pressure at home?

If they already monitor – can you tell me a bit more about this?

Can you tell me about what it's like for you taking medication for your high blood pressure?

Can you tell me how you feel about your GP making changes to your blood pressure medication?

HOME BP study experience

Can you tell me why you signed up for the Home BP study?

Can you tell me all about what it has been like for you being in the HOME BP study in the usual care group?

At the start of the HOME BP study, you had a baseline appointment with a nurse at your GP Practice – can you tell me about that?

How did you find it using the HOME BP programme to fill in your questionnaires online at the start of the study?

For peer review only

After you had completed your questionnaires, did you have an appointment with your GP to check your blood pressure medication? Can you tell me about this?

HCP support

Can you tell me about the contact you have had with your GP or nurse so far in the HOME BP study?

Can you tell me about how you have found the support from your GP or nurse while you were taking part in the HOME BP study?

For peer review only

BMJ Open

A qualitative process study to explore the perceived burdens and benefits of a digital intervention for self-managing high blood pressure in Primary Care in the UK.

Journal:	*BMJ Open*
Manuscript ID	bmjopen-2017-020843.R2
Article Type:	Research
Date Submitted by the Author:	28-Feb-2018
Complete List of Authors:	Morton, Katherine; University of Southampton, Psychology<br>Dennison, Laura; University of Southampton, Psychology<br>Bradbury, Katherine; University of Southampton, Psychology<br>Band, Rebecca; University of Southampton, Psychology<br>May, Carl; University of Southampton, Faculty of Health Sciences<br>Raftery, James; Southampton University, School of Medicine<br>Little, Paul; University of Southampton, Primary Care and Population Science<br>McManus, Richard; University of Oxford, Dept of Primary Care Health Sciences<br>Yardley, Lucy ; University of Oxford, Nuffield Department of Primary Health Care Sciences
**Primary Subject Heading**:	Qualitative research
Secondary Subject Heading:	Cardiovascular medicine, Health services research, Public health
Keywords:	Self-management, digital intervention, qualitative, treatment burden

SCHOLARONE™
Manuscripts

Title page

A qualitative process study to explore the perceived burdens and benefits of a digital intervention for self-managing high blood pressure in Primary Care in the UK.

Kate Morton[a]*, Laura Dennison[a], Katherine Bradbury[a], Rebecca Band[a], Carl May[b], James Raftery[c], Paul Little[d], Richard J. McManus[e], & Lucy Yardley[a]

a. Academic unit of psychology, University of Southampton, Southampton, UK
b. Faculty of Health Sciences, University of Southampton, Southampton, UK
c. Faculty of Medicine, University of Southampton, Southampton, UK
d. Primary Care Research, University of Southampton, Southampton, UK
e. Nuffield Department of Primary Care Health Sciences, University of Oxford, Oxford, UK

*Corresponding author: ksm1r13@soton.ac.uk; 02380 592234

Room 3063, B44, University of Southampton, Southampton, SO17 1BJ

Word count 5044

For peer review only - http://bmjopen.bmj.com/site/about/guidelines.xhtml

ABSTRACT

Objectives: Digital interventions can change patients' experiences of managing their health, either creating additional burden or improving their experience of healthcare. This qualitative study aimed to explore perceived burdens and benefits for patients using a digital self-management intervention for reducing high blood pressure. A secondary aim was to further our understanding of how best to capture burdens and benefits when evaluating health interventions.

Design: Inductive qualitative process study nested in a randomised controlled trial.

Setting: Primary Care in the UK

Participants: 35 participants taking antihypertensive medication and with uncontrolled blood pressure at baseline participated in semi-structured telephone interviews.

Intervention: Digital self-management intervention to support blood pressure self-monitoring and medication change when recommended by the healthcare professional.

Analysis: Data were analysed using inductive thematic analysis with techniques from grounded theory.

Results: Seven themes were developed which reflected perceived burdens and benefits of using the intervention, including worry about health, uncertainty about self-monitoring, and reassurance. The analysis showed how beliefs about their condition and treatment appeared to influence participants' appraisal of the value of the intervention. This suggested that considering illness and treatment perceptions in Burden of Treatment theory could further our understanding of how individuals appraise the personal costs and benefits of self-managing their health.

For peer review only

For peer review only - http://bmjopen.bmj.com/site/about/guidelines.xhtml

Conclusions: Patients' appraisal of the burden or benefit of using a complex self-management intervention seemed to be influenced by experiences within the intervention (such as perceived availability of support) and beliefs about their condition and treatment (such as perceived control and risk of side effects). Developing our ability to adequately capture these salient burdens and benefits for patients could help enhance evaluation of self-management interventions in the future. Many participants perceived important benefits from using the intervention, highlighting the need for theory to recognise that engaging in self-management can include positive as well as negative aspects.

Trial registration: ISRCTN13790648. Registered 14 May 2015.

Keywords: Self-management; digital intervention; qualitative; treatment burden

Strengths and limitations of this study:

- The exploratory, open approach to data collection enabled us to capture whichever benefits or burdens were most salient to the participants.
- We only interviewed participants at one point in time, so were unable to gain an understanding of dynamic changes in perceived benefits or burdens over time.
- Both well and poorly controlled hypertensive patients took part in the interviews, but it was difficult to recruit low users of the intervention which could limit the generalisability of the findings.
- The asymptomatic nature of hypertension and the unique medication change pathway means that these findings may not be generalisable across conditions.

For peer review only

For peer review only - http://bmjopen.bmj.com/site/about/guidelines.xhtml

BACKGROUND

The work involved in looking after one's health when living with a chronic condition can include complex tasks such as organising and adhering to treatment regimens, interacting with healthcare professionals (HCPs), regular monitoring of health indicators, and making health-related decisions, all of which can accumulate into a considerable burden [1]. Digital self-management interventions are often developed to improve health outcomes, but these interventions could also either increase or minimise the burden of the healthcare process for patients. Developing our understanding of the burdens of self-management can help to better optimise the delivery of healthcare to improve adherence and well-being[1-3]. Burden of Treatment (BoT) theory provides a mechanism for understanding these experiences in the context of patients' personal capacity to cope, with emphasis on the role of wider healthcare systems and social networks available to the patient[1].

Health economic evaluations also focus on understanding the impact of healthcare on patients, seeking to weigh up the resources used against the health outcomes in order to better inform decision-making. Recent guidelines for economic evaluations in health and medicine recommend adopting a societal perspective such that all relevant outcomes are evaluated, rather than focusing only on formal healthcare costs[4]. In particular, personal costs such as time spent in self-care should be included. Consequently, BoT theory and health economic evaluations share an interest in adequately capturing the wider burdens or personal costs of engaging with healthcare. For consistency in terminology in this paper, negative outcomes/personal costs of healthcare will be referred to as 'burdens'.

BoT theory considers patients' time as a resource that is used by the healthcare system, while health economic evaluation counts time as an 'opportunity cost' whereby the patient 'spends' time that could have been spent on something other than healthcare. However, subjective experiences of time spent on digital interventions may be varied and complex. Heterogeneity in the relative value placed on the outcomes of the intervention[5] may mean that for some participants the time spent

For peer review only - http://bmjopen.bmj.com/site/about/guidelines.xhtml

engaging with elements of an intervention is not perceived as a burden but rather as a benefit, either because it is interesting, pleasant or meaningful in and of itself or because of the positive outcomes it can lead to. In other words, some people may actually like engaging with healthcare. The value of exploring the personal benefits of intervention participation has not received as much focus as understanding the costs, such as treatment burden. McNamee et al.[6] proposed that the health research guidelines for economic analysis may need to be adjusted for digital health interventions to ensure we can fully capture the heterogeneous costs and benefits arising when complex interventions are implemented in complex systems.

To further our understanding of how patients perceive benefits and burdens when using digital health interventions, we carried out a qualitative process study [7]. The digital HOME BP intervention was developed based on best practice recommendations to help improve hypertension in poorly controlled patients by facilitating self-monitoring of blood pressure (BP) at home and prompting appropriate intensification of medication by HCPs[8]. This intervention could help to minimise the treatment burden of hypertension by providing an online healthcare system in which HCPs have sight of patients' home readings, streamlining the process for finding the most effective medication without the need for attending the GP surgery. However, HOME BP is a complex, interactive multi-component intervention, which creates potential diversity in the perceived burden and benefits for participants using it. The contexts in which the intervention is embedded may also be diverse, and factors such as individual differences in patients' health status, beliefs about medication and risks of high BP, availability of time and resources, and access to support may influence how the intervention is perceived and valued. The HOME BP intervention was developed using the person-based approach[9] which emphasises the importance of understanding participants' unique perspectives and different situations when developing and implementing digital interventions. Adopting a more granular approach to the evaluation of benefit and burden is consistent with the

For peer review only - http://bmjopen.bmj.com/site/about/guidelines.xhtml

person-based approach, and with the BoT approach of fully understanding the participants' perspective.

The present study aimed to explore the perceived burden and benefits of using a digital health intervention for self-managing BP using qualitative process interviews with intervention and usual care participants taking part in a randomised controlled trial (RCT).This paper seeks to interpret the implications for optimising the capture of perceived costs and benefits in health economic evaluations and evaluating the burden of treatment.

METHODS

Design

A qualitative process study embedded in the HOME BP trial[8] was approved by the University of Southampton and NHS Research Ethics committees. The COREQ checklist (Consolidated criteria for reporting qualitative studies) was used to ensure comprehensive reporting of the study[10] (supplementary file 1).

Intervention

The HOME BP programme supported participants to self-manage their high BP, primarily via home self-monitoring of BP and making changes to dose/drug type when recommended by the HCP. Lifestyle change modules were also available, but optional as the key target behaviours for the intervention were self-monitoring and medication change adherence [8 11]. Participants using HOME BP were supported by a 'prescriber' (GP or nurse prescriber responsible for changing medication)

For peer review only - http://bmjopen.bmj.com/site/about/guidelines.xhtml

and a ‘supporter’ (nurse or healthcare assistant who supported participants in self-monitoring and choosing lifestyle changes).

Participants were invited to use the online programme by their GP and were randomised to usual care or intervention after completing baseline measures online. Those randomised to the intervention group completed two online training sessions which sought to overcome concerns about variability in readings and changing medication. Participants were encouraged to monitor their BP in the mornings, but the programme allowed flexibility as it was most important that people found a time of day that suited them to monitor their BP. Both intervention and usual care participants were followed up at 6 and 12 months post-randomisation.

Table 1 describes the HOME BP intervention in more detail.

Table 1 HOME BP Intervention Characteristics

Target behaviour	Description
Self-monitoring BP	Participants monitored their BP at home for 7 days every 4 weeks. After 7 days, they entered their BP readings on the HOME BP website and received instant automated feedback using a traffic light system. If BP was very high (red) or very low (blue), they were told to contact their GP surgery. If BP was above target (amber), they were told their prescriber would contact them about a medication change. If BP was on target (green), they were congratulated and asked to monitor their BP again next time.
Medication change	The prescriber planned three potential medication changes with the participant at the start of the study. HOME BP informed prescribers by email when a patient’s home BP readings were above-target and they could implement a pre-

For peer review only

For peer review only - http://bmjopen.bmj.com/site/about/guidelines.xhtml

	planned change without needing to see the participant for an appointment.
Optional lifestyle changes	At nine weeks after randomisation, participants had the option of choosing an online session to support lifestyle change to help control their BP, specifically weight management, salt reduction, healthy diet, physical activity, or alcohol reduction. Participants were alerted by email when this became available, and saw an option to view the healthy lifestyles session each time they logged on to HOME BP. The online lifestyle change sessions could be started at any time during the 12-month trial, from nine weeks.

For peer review only

Participants

Patients were eligible to take part in the HOME BP trial if they had uncontrolled hypertension managed in Primary Care (mean BP reading of 140/90 mm Hg or more at baseline taken at the GP surgery using a validated electronic automated sphygmomanometer (BP TRU BPM 200)). In addition, they needed to be prescribed 1-3 antihypertensive medications at baseline, and aged over 18 (full inclusion and exclusion criteria are listed in the protocol[8]).

Both intervention and usual care participants were invited to take part in interviews as we felt that obtaining an understanding of managing BP in usual care would aid interpretation of the perceived burden and benefits of the intervention. We aimed to speak to participants at a range of time-points during the 12-month trial from 10 weeks onwards as this gave participants the opportunity to become familiar with HOME BP. No new intervention content was introduced after the lifestyles sessions became available at nine weeks.

For peer review only - http://bmjopen.bmj.com/site/about/guidelines.xhtml

Recruitment and interview procedure

A sub-sample of RCT participants were invited by email to provide feedback on their experiences of managing their BP (n=78, of 622 patients in the RCT). Informed consent was taken by post or online, depending on participant preference. Recruitment was initially opportunistic, but subsequently a purposive approach was adopted to target younger participants, low engagers, and those with recent uncontrolled self-monitored BP readings, informed by the concurrent analysis. Recruitment was stopped once the researchers agreed that data saturation had been reached and no new burdens or benefits were arising.

Semi-structured interview schedules were co-developed by experts in health psychology (KM, KB, RB, LY, LD), health economics (JR) and sociology (CM). Open, inductive questions were carefully selected to elicit data about the burden and benefits of BP management perceived as most salient by the participants (see Supplementary File 2 for interview schedules). The interviews were conducted by telephone to minimise the burden on participants, except in one case where the participant asked to meet face-to-face due to struggling with hearing on the telephone. The interviews took place between February 2016 and February 2017. Each participant was given a £10 gift voucher to thank them for their time.

All interviews were conducted by KM (MSc, BSc. termed “the researcher”), a female PhD candidate in Health Psychology who was also employed as a research assistant. Each interview was audio-recorded, and the researcher also took notes and completed a self-reflection log afterwards to record any emerging thoughts on the data. Audio-recordings were transcribed verbatim and checked thoroughly by the researcher.

Patient and Public Involvement

Patient and public involvement (PPI) representatives have been involved in the design and conduct of the randomised controlled trial, including decisions about recruitment processes, outcome

For peer review only

For peer review only - http://bmjopen.bmj.com/site/about/guidelines.xhtml

measures and trial procedures. We also discussed the findings of this qualitative process study with our PPI to facilitate our interpretations of the data. The participants in the study were patients, ensuring we were collecting experiences of burden from the target population, and the results were fed back to the study participants as a newsletter.

Analysis

The analysis was an iterative process led by KM, supported by frequent discussion of emerging themes with LY and LD (who have extensive experience in qualitative research) along with input regarding health economic and sociological perspectives (JR and CM). Inductive thematic analysis methods were used[12 13] with techniques from grounded theory such as memoing, constant comparison, and diagramming to enhance our understanding and facilitate the development of higher themes[14 15]. Data collection and analysis ran concurrently to enable purposive sampling based on analytic insights. Thorough line-by-line coding was undertaken in NVivo 10[16], and a coding manual was developed which evolved as more data were collected and coded. The emerging codes were constantly checked against the raw data to ensure the analysis was driven by the participants' own language and experiences.

All data relating to burdens and benefits of managing BP were analysed. We also coded factors that appeared to influence perceptions of burdens and benefits to facilitate an in-depth understanding of how participants appraised the intervention's value. A broad and open definition was adopted whereby benefits and burdens were defined as positive and negative outcomes or experiences of engaging in the intervention[17], in order to facilitate a comprehensive representation of all potentially relevant data.

For peer review only - http://bmjopen.bmj.com/site/about/guidelines.xhtml

RESULTS

Participant characteristics

In the intervention group, 28 of 54 invited participants agreed to be interviewed (52%). In the usual care group, 7 of 24 invited participants agreed (29%). Most participants who did not take part chose not to reply, but those who did said they did not have anything to report on the trial (n = 3 in usual care). The participants were from 19 different GP surgeries. Table 2 shows the sociodemographic and intervention details of the sample.

Table 2. Sociodemographic and intervention participant data (n=35)

	Intervention participants	Usual care participants
N	28	7
Median duration of interview (range)	38 (15-67) minutes	28 (22-40) minutes
Median age (range)	70 (41-87) years	67 (52-77) years
Gender	71% female	43% female
Ethnicity		
White	24	6
Black African	1	
Pakistani	1	
Other	2	1
Education levels	9 No formal education	2 No formal education
	8 GCSE or A-level	3 GCSE or A-level

For peer review only

For peer review only - http://bmjopen.bmj.com/site/about/guidelines.xhtml

	10 Higher Education	1 Higher Education
	1 Other	1 Other
Median number of weeks into the study at which the interview took place (range)	20 (10-57) weeks	17 (7 to 24) weeks
Poorly controlled BP at the time of the interview	10/28 (36%)	N/A*
Medication change recommended during the study	15/28 (54%)	N/A
Accessed optional healthy lifestyles session	15/28 (54%)	N/A

212 *As BP self-monitoring was a key component of the intervention, BP readings were available for the
213 intervention group throughout the duration of the study but data about BP from the usual care
214 group were only available at RCT baseline and follow-up points.

215 **Themes**

216 Table 3 presents seven themes exploring perceived burdens and benefits of the HOME BP
217 intervention. One meta-theme also emerged concerning how illness and treatment beliefs about
218 high BP appeared to influence participants' perceptions about the intervention's burdens and
219 benefits, and this is discussed in relation to each theme it applies to. Figure 1 shows how illness and
220 treatment perceptions about BP appeared to relate to the sub-themes identified by the thematic
221 analysis.

222

For peer review only - http://bmjopen.bmj.com/site/about/guidelines.xhtml

Where quotes are included, participants are referred to as 'p' followed by a number. Study group (intervention or usual care) is also included to help understand the quotes in context.

For peer review only

Table 3. Themes and sub-themes relating to perceived burdens and benefits of the intervention

Themes	Sub-themes	Exemplar participant quote
Benefit of reassurance from seeing BP readings	Reassurance when BP readings are well-controlled	"I'm so pleased. And my mind is at rest when we go on holidays and all that...I'm alright. I'm alright sort of thing. Yeah, peace of mind" (Intervention p9, well-controlled)
	Reassurance from keeping an eye on BP	"It made me much more aware of what the problem is with the high blood pressure and by monitoring it so regularly, I know exactly where I stand with it" (Intervention p15, well-controlled)
Benefit of motivation for lifestyle change from seeing BP readings	Seeing BP readings motivated lifestyle change	"It is quite interesting to see the effects of what I'm doing on the blood pressure and everything. So, I think that is – it is quite good" (Intervention p18, well-controlled)
Benefit of better	Perceived health improvements	"It helped me to change my medication and then because of change of medication,

For peer review only

health	from medication changes	my blood pressure went down. So definitely there is a benefit" (Intervention p16, well-controlled)
	Intervention can facilitate management of side effects	“That medication didn’t work, in that I was on holiday and my ankles swelled up so much – and my feet and my legs, so much so that I couldn’t see my toes. So I stopped taking that medication. Was called back to the GP. And I’m now on a medication that works for me and is managing the blood pressure” (Intervention p7, well-controlled)
Burden of worrying about health	Negative emotional responses to seeing high readings	"I was actually quite shocked because it was a—a lot higher" (Intervention p6, poorly controlled)
	Worrying about medication change affecting health	"I don’t want to get more medication ‘cause I’m already on a high dose and I don’t want to increase it because it worries me about my kidneys" (Intervention p24, poorly controlled)
Burden of uncertainty from self-monitoring	Uncertainty about whether readings are representative	"If someone only ever takes it in the morning, and you tend to get those lower readings, are you really getting a true picture of what they’re like in the afternoon or the evening?" (Intervention p10, well-controlled)

For peer review only - http://bmjopen.bmj.com/site/about/guidelines.xhtml

	Uncertainty about what to do about high or low readings	"I don't know what's going to happen in respect to that [amber feedback]. Whether I'm going to get a call from my GP, or whether he – so I'm a little bit, like, you know, in the air. I don't really know what's going to happen in that respect" (Intervention p22, poorly controlled)
Burden of thinking about making healthy lifestyle changes	Worry or guilt about not engaging with healthy changes	"I have looked at it [online healthy lifestyles session]. I wouldn't say I've looked at it seriously, and I need to" (Intervention p4, poorly controlled)
Burden of the practicalities of adhering to intervention procedures	Burden of fitting self-monitoring into the day	"I like to get up and have a cup of coffee and I'm thinking 'Well, let's get the blood pressure done first because otherwise I can't do that, you know, for a while afterwards.' So, I've found that quite—quite difficult" (Intervention p5, poorly controlled).

226

For peer review only - http://bmjopen.bmj.com/site/about/guidelines.xhtml

For peer review only

For peer review only - http://bmjopen.bmj.com/site/about/guidelines.xhtml

Insert Figure 1 here

Benefit of reassurance from seeing BP readings

Reassurance when BP readings are well-controlled

Seeing well-controlled readings when self-monitoring BP gave participants peace of mind which was widely perceived as a benefit of the intervention. People described feeling relieved that their BP readings were lower than at the GP surgery, and felt this gave them more insight into what their BP was like most of the time.

“What I do like about it is taking the blood pressure here at home, the readings are lower. And I find that quite reassuring that my blood pressure is not always high” (Intervention p11, well-controlled)

Several usual care participants had decided to use their own BP monitors, and this group also described feeling reassurance when seeing their BP was well-controlled.

Reassurance from keeping an eye on BP

Most participants liked having an increased focus on their BP through regular monitoring and found it interesting to compare their readings over time. However one participant perceived that taking BP regularly could encourage too much attention on your health, which was a potential burden of the intervention for her (Intervention p28, BP control unknown as did not enter BP readings on HOME BP). This participant had low concern about her BP generally, and was not motivated to engage in self-management.

Even when participants had poorly-controlled readings, many felt a benefit from the intervention as it enabled them to regularly check their BP and detect any problems instantly rather than carrying on unaware.

For peer review only

"I think it's helping me to know where my blood pressure stands because it's a regular thing every month" (Intervention p24, poorly-controlled)

The knowledge that home readings were shared with the prescriber reassured participants as they knew that any problems would not only be detected but also dealt with at the time, making them feel well cared for. This contrasted with the perceived burden of managing BP in usual care where some participants felt concerned that their GP did not change their medication when their home readings were too high, or would have liked more regular contact with their GP surgery to check their BP and medication.

"It would be nice to have it checked, I guess, you know, every three months or whatever. How— however often. I mean, how do they know that everything is working?" (Usual care p4).

This shows that although participants in usual care gained reassurance from seeing low readings when they monitored at home, the lack of interaction with the GP surgery could cause concern when readings were high or when patients did not regularly monitor BP at home of their own accord.

Benefit of motivation for lifestyle change from seeing BP readings

Some participants were motivated to increase their physical activity, engage in stress management activities or healthy eating because they could see this had a positive impact on their BP readings. This helped them feel more in control of their BP.

"By taking the readings regularly and frequently, it gave me more of a feedback straightaway if you like about anything, changes that I did make like a bit of exercise or…practicing relaxation and this sort of thing. So that was quite nice, it was nice to feel that I was more in control of it again" (Intervention p20, well-controlled)

For peer review only

For peer review only - http://bmjopen.bmj.com/site/about/guidelines.xhtml

Other participants felt frustrated after making lifestyle changes in the past which had no effect on their BP. This made them feel that lifestyle was ineffective for controlling BP.

"I'm a completely different person. My diet's completely different. And my blood pressure remained the same. So I've done literally everything you physically possibly can to help yourself, and nothing's worked" (Intervention p1, well-controlled).

Benefit of better health

Perceived health improvements from medication changes

Many participants felt it was beneficial to change their medication when their readings were too high, and were very pleased when they perceived that a medication change led to lower BP readings because of the positive effect this would have on their health.

"I've found that by having the medication changed up at regular intervals my blood pressure's improved all the time" (Intervention p15, well-controlled)

A few participants felt that a medication change had not been effective at lowering their BP which could create doubt about their medication's effectiveness.

"It's been doubled but it hasn't seemed to lower my blood pressure at all, in fact, it's at the same levels as it is sort of now, un-medicated. So I just think – I don't think it's the right one. You know, I can take the tablet but, actually, I don't think it's doing anything". (Intervention p26, poorly-controlled)

Intervention can facilitate management of side effects

Most participants did not experience any side effects from having their medication changed. Where side effects did occur, participants tended to perceive this as being a cost of taking medication

For peer review only - http://bmjopen.bmj.com/site/about/guidelines.xhtml

(which was balanced against the benefit of controlling BP), rather than a burden of the intervention itself. They felt that the intervention could help them to be more aware of side effects, to identify alternative medications and to monitor how these affect their health.

"That [side effect] would have happened, you know, no matter what. That would have been an issue but this has actually highlighted it, sort of, more clearly" (Intervention p5, poorly-controlled)

Burden of worrying about health

Negative emotional responses to seeing high readings

A burden of self-monitoring BP for some people was that seeing high readings could cause worry about health. Participants' beliefs about their BP control appeared to influence their appraisal of high readings. A few participants believed their BP was well-controlled, a belief which was perhaps reinforced by clinical staff approving their readings previously, and had only joined the study to help with research. These participants tended to feel shocked or annoyed when they received above-target feedback from the intervention as this challenged their beliefs.

"At one time, I was told to go on medication, further medication, which I must admit I was not very happy about… When I used to go for a check with the nurse, if I'd have had those particular readings, they wouldn't have been high" (Intervention p17, poorly controlled)

Others were confused or frustrated by high BP readings when they could not understand why this might have happened.

"I'm thinking about why my blood pressure has gone up. I can't think why" (Intervention p25, poorly controlled).

For peer review only - http://bmjopen.bmj.com/site/about/guidelines.xhtml

Meanwhile people who expected to see high readings were less concerned because they had accepted that high readings were likely.

"Just par for the course. It's what I expect from my blood pressure, really, so, it never worries me" (Intervention p5, poorly controlled)

Perceptions about the causes of high BP also influenced how anxious people felt about seeing high readings. Those who felt that high readings held serious implications for their health tended to feel frightened. Some even felt apprehensive *before* self-monitoring in case their readings were out-of-range, as they didn't want to see evidence that their BP was too high or low.

"Before I take my blood pressure, I do get stressed. I wouldn't say I get massively stressed because obviously I'm used to doing it now but … it's just that apprehension and thinking 'Oh, God, I hope it's not too high today. I wonder really what's going on and how serious this is". (Intervention p26, poorly controlled).

Other people were able to dismiss one-off high readings without feeling anxious as they attributed high readings to less threatening explanations such as feeling stressed, not sitting still for long enough, positioning of the cuff, or held a prior expectation of it being normal for BP to fluctuate. In these cases, the high readings had less negative emotional impact as they were not interpreted as indicating a serious underlying health issue.

Worrying about medication change affecting health

Some participants were worried about the effects that changing BP medication could have on their health. Previous experience of side effects, existence of co-morbidities, and concerns about medication dependency or impact on kidneys tended to make participants feel more worried about changing medication.

For peer review only - http://bmjopen.bmj.com/site/about/guidelines.xhtml

Perceptions about the health risk of high BP in terms of stroke and cardiovascular disease tended to affect how burdensome participants perceived a medication change to be. Anxiety about future health could override concerns about medication side effects or dependency as the behaviour was evaluated as beneficial in order to bring BP down, although sometimes participants still experienced conflict between the perceived benefit and burden.

"The blood pressure has gone down but now my worries have changed from blood pressure to other things. One is actually depending on medicine whole of my life. And secondly impact of medicine on my body like kidneys" (Intervention p16, well-controlled).

Burden of uncertainty from self-monitoring

Uncertainty about whether readings are representative

Whilst some participants were confident making decisions about when to monitor their BP, others were worried about whether their readings were representative, especially when BP was seen to vary at different times of day or after physical activity or drinking coffee. This could lead to doubt about the meaningfulness of self-monitoring and the recommendations of the intervention.

"I wonder if maybe the time of day I'm doing it, maybe my blood pressure's always gonna be roughly that. And could it be different during the day, is the sort of thing that does play in my mind a bit" (Intervention p1, well controlled).

Uncertainty about what to do about high or low readings

Uncertainty could also become a burden after seeing an out-of-range BP reading, as the participant had to decide what to do next. This burden was removed when the prescriber provided quick, personalised feedback to the participant, but when they did not receive any contact from their

For peer review only

For peer review only - http://bmjopen.bmj.com/site/about/guidelines.xhtml

prescriber or felt the prescriber was not available to provide support, this could create a feeling of doubt.

"I suppose I knew there was nothing to worry about but it's always a bit of a niggle in the back of your mind… even the days she's [the nurse prescriber] at work I can't ring her at work because she may be, you know, doing something else" (Intervention p21, well-controlled)

Burden of thinking about making healthy lifestyle changes

Worry or guilt about not engaging with healthy changes

Several participants felt they would like to lose weight, eat more healthily, or do more physical activity but lacked the motivation or self-efficacy to make these changes, especially if they had other co-morbidities. This could create feelings of guilt or worry about their failure to make healthy changes, which was a burden of the intervention for them.

"I understand that, obviously, I need to get my blood pressure down because it is very dangerously high, but I just don't know what to do about it, you know?... where I feel fatigued and worn out, I don't feel well enough at the moment to do any exercise" (Intervention p26, poorly controlled)

Burden of the practicalities of adhering to intervention procedures

Burden of fitting self-monitoring into the day

Many participants felt that self-monitoring was easy to fit into their day, and some described this as being easier than going to the GP surgery to have their BP taken. Those with busy daily lifestyles tended to find it harder to remember to self-monitor, and a burden for some participants was

For peer review only

For peer review only - http://bmjopen.bmj.com/site/about/guidelines.xhtml

deciding how best to fit self-monitoring into their routine given the instructions about not drinking coffee or exercising beforehand.

The perceived burden of regular self-monitoring seemed to be mitigated by the perceived benefit of the behaviour, such that those who felt reassurance from seeing low readings or with high motivation to control BP found it less hassle and easier to remember than those who felt anxious about self-monitoring or had only joined the study to help with research.

"There was no big deal. It doesn't take long and it's—it's quite nice to sit down and have a relax during the day" (Intervention p8, well-controlled)

For peer review only

For peer review only - http://bmjopen.bmj.com/site/about/guidelines.xhtml

389 **DISCUSSION**

390 This qualitative study has identified diverse perceived burdens and benefits of using a self-
391 management digital intervention for high BP. In support of the BoT theory[1], the HOME BP
392 intervention appeared to reduce the burden on patients to self-manage their condition by improving
393 access to regular HCP support and facilitating better understanding of their condition, but in some
394 cases there was a burden of worry about health or changing medication. How much benefit a
395 patient perceived from the intervention compared to burden seemed to be influenced by the
396 dynamics of the patient-HCP interaction (described as 'Improving Cooperation" in BoT theory) and
397 the patient's own resources to manage their condition and cope with medication (described as
398 "Capacity").

399 Another important factor relating to the burden experienced was personal beliefs about BP and
400 treatment. Those who recognised that their BP was too high and did not have concerns about side
401 effects or taking medication appeared to have more positive experiences of the intervention,
402 perceiving self-monitoring as more worthwhile, and feeling less anxious about seeing high readings
403 or changing medication. This is consistent with the necessity-concerns framework[18]. BoT theory
404 states that people who are better equipped with resources and are more resilient may cope better
405 with the burden imposed by healthcare[19], but the importance of an individual's personal
406 conceptualisation of their condition in how burdensome they find self-care is not strongly
407 represented. This beliefs system may be partly encompassed by the "Relational Integration" aspect
408 of BoT theory, which refers to the extent to which patients trust the tasks they do for healthcare, e.g.
409 self-monitoring BP, and feel confident in the outcomes of these tasks, e.g. changing medication.
410 However illness and treatment perceptions[20] are not explicitly covered by the theory and it may be
411 helpful to consider them as additional factors which might influence the experience of treatment
412 burden.

For peer review only - http://bmjopen.bmj.com/site/about/guidelines.xhtml

Implications for measurement of benefit and burden

The present study demonstrates the value of collecting in-depth qualitative data to develop a detailed understanding of the burden of treatment, and to discover perceptions specific to the context in which the intervention was implemented. The important psychosocial outcomes discovered using qualitative research can inform the selection or development of relevant quantitative measures to capture these factors in further evaluation.

Quantitative measures have been developed to appraise the structural aspects of burden of treatment[21 22], but these are not intended to assess psychosocial factors such as reassurance, anxiety or uncertainty which this study suggests can influence the extent to which using an intervention is experienced subjectively as a burden.

Future research could explore how best to capture the perceived burden or benefit of an intervention. One approach might be to simply ask participants to quantify the net subjective burden or benefit of interventions. However, it could be challenging for participants to weigh complex heterogeneous psychosocial outcomes against one another and decide overall whether an intervention was more burdensome or beneficial. Capturing the extent to which patients experience positive or negative psychosocial outcomes might better assess how beneficial or burdensome the intervention was perceived to be. Although this would not produce a single outcome measure, cost-consequence analysis can be used to inform decision-making when an intervention has multiple relevant outcomes which cannot be aggregated into one value[23]. Coast[24] discusses whether a multidimensional approach is more informative for economic analysis or if a single aggregated value is more pragmatic.

Extending the evaluation of outcomes beyond health is in line with the capability approach[25], which focuses on broader aspects of subjective well-being which are not assessed by generic measures

For peer review only - http://bmjopen.bmj.com/site/about/guidelines.xhtml

such as the EQ-5D[26]. Tools used to capture perceived capability (such as the ICECAP[27], and ASCOT[28]) are gaining support as holistic measures of economic evaluation, but do not assess the more specific psychosocial burdens and benefits of healthcare raised by participants in this study. Process utility emphasises the need to quantitatively measure the value that people attach to healthcare delivery. This approach might be relevant for evaluating how much value people perceive in the process of using digital health interventions and the capability this achieves[29]. It has been argued that process utility measures should also ask about the reasons behind patients' valuations, to better inform the decision-maker[30]. This would help to capture the individual differences found in this study in how people appraise the personal value of a digital intervention, informed by their underlying illness and treatment beliefs.

Strengths and limitations

A strength of the study was that we used relatively open questions formulated by a multi-disciplinary team which enabled us to elicit and explore a wide range of perceived burdens and benefits, some of which were not anticipated at the outset of research. We are aware of the lead researcher's potential influence on the data analysis, which we strived to minimise by transparent memoing of decisions and regular team meetings to discuss the emerging themes. Participants were sent newsletters to describe the findings of the study, but were not invited to provide feedback on the analysis.

We succeeded in speaking to well and poorly controlled hypertensive participants at different points in the intervention, and there was a wide range of demographics in terms of age, education level and gender in the sample. However, the uptake rate from those invited to interviews was not high, particularly in the usual care group. Perhaps unsurprisingly, it was difficult to recruit low engagers in the intervention group, which could have helped reach theoretical saturation. In terms of wider applicability, we are aware that these findings may not be generalisable across other health

For peer review only - http://bmjopen.bmj.com/site/about/guidelines.xhtml

conditions, as the lack of symptoms in hypertension and the stepped pathway for changing medication are quite unique features of this condition.

Repeated interviews with the same participants may have offered more insight into the dynamic nature of perceived burdens and benefits over time, although more regular conversations about the target behaviour could have influenced participants' BP management behaviour therefore threatening the RCT conclusions. It has been noted that a key issue with process evaluations of interventions is the tendency for intervention content and impact to change over time[7], such that deciding the optimal point to collect evaluation data is challenging.

Some of the burdens and benefits described by patients in this study were also found to a lesser extent in the qualitative development of the HOME BP intervention, such as reassurance from seeing well-controlled readings, and some concerns about side effects and high or variable readings [31]. Others were novel and only arose when participants experienced the full HOME BP intervention during the RCT as opposed to a prototype, for example the perceived health improvements from medication changes. This demonstrates the value of conducting inductive qualitative research to explore users' perspectives at each stage of intervention development and evaluation, in line with the person-based approach[9].

CONCLUSIONS

In the context of this digital intervention, the study shows that participants' appraisal of burdens and benefits appeared to be influenced by both intervention factors, such as BP readings and perceived availability of the healthcare professional, and patient characteristics, such as perceptions of BP control, previous experience of side effects, and co-morbidities. This nuanced evaluation would be lost in a population-level analysis, demonstrating the advantage of a more individualised approach

For peer review only - http://bmjopen.bmj.com/site/about/guidelines.xhtml

for better understanding participants' perspectives of an intervention and how best to minimise the burden of treatment.

The study develops the recommendations of McNamee et al [6] that complex digital health interventions warrant a wider perspective for measuring health outcomes, and discusses the implications of capturing broader psychosocial outcomes for Burden of Treatment theory and health economic evaluations.

The finding that some participants perceived personal benefits from using the intervention demonstrates that the process of healthcare can, in itself, be positive for some people, highlighting the importance of capturing transient short-term benefits to take these into account as well as the burden of self-management.

For peer review only

For peer review only - http://bmjopen.bmj.com/site/about/guidelines.xhtml

CONTRIBUTORS

KM recruited participants, conducted interviews, analysed data, and wrote manuscript. LD & KB contributed to study design and data analysis. KB and RB developed the digital intervention. PL & RJM contributed to intervention development and interpretation of themes. CM & JR contributed to theoretical and methodological implications of the study findings. LY contributed to study design, data collection, data analysis, and interpretation.

All authors contributed to the manuscript preparation and provided final approval of the version to be published.

ACKNOWLEDGEMENTS

We would like to thank all the participants who took part in this research, and the PPI who helped in the design and conduct of the research.

FUNDING

This independent research was funded by the National Institute for Health Research (NIHR) Programme Grants for Applied Research Programme (Grant Reference Number RP-PG-1211-20001). The views expressed are those of the author(s) and not necessarily those of the NHS, the NIHR, or the Department of Health. RJM is funded by an NIHR Professorship (NIHR-RP-R2-12-015) and the NIHR Oxford CLAHRC.

COMPETING INTERESTS

RJM has received BP monitors for research purposes from Omron and Lloyds Pharmacies.

ETHICS APPROVAL

This qualitative process study was embedded in the HOME BP trial and approved by the University of Southampton and NHS Hampshire A Research Ethics committees (REC Reference 15/SC/0082).

For peer review only - http://bmjopen.bmj.com/site/about/guidelines.xhtml

DATA SHARING STATEMENT

Requests for data sharing can be sent to the corresponding author. Full transcripts of interviews are not available to protect participants' anonymity.

Figure legend:

Figure 1 Possible influences of illness and treatment beliefs on perceived burdens and benefits of the intervention

For peer review only

For peer review only - http://bmjopen.bmj.com/site/about/guidelines.xhtml

REFERENCES

1. May CR, Eton DT, Boehmer K, et al. Rethinking the patient: using Burden of Treatment Theory to understand the changing dynamics of illness. *BMC Health Serv Res* 2014;**14**(1):281.

2. Eton DT, de Oliveira DR, Egginton JS, et al. Building a measurement framework of burden of treatment in complex patients with chronic conditions: a qualitative study. *Patient Relat Outcome Meas* 2012;**3**:39.

3. Gallacher K, May CR, Montori VM, et al. Understanding patients' experiences of treatment burden in chronic heart failure using normalization process theory. *Ann Fam Med* 2011;**9**(3):235-43.

4. Sanders GD, Neumann PJ, Basu A, et al. Recommendations for conduct, methodological practices, and reporting of cost-effectiveness analyses: second panel on cost-effectiveness in health and medicine. *JAMA* 2016;**316**(10):1093-103.

5. Ioannidis JP, Garber AM. Individualized cost-effectiveness analysis. *PLoS Med* 2011;**8**(7):e1001058.

6. McNamee P, Murray E, Kelly MP, et al. Designing and undertaking a health economics study of digital health interventions. *Am J Prev Med* 2016;**51**(5):852-60.

7. Moore GF, Audrey S, Barker M, et al. Process evaluation of complex interventions: Medical Research Council guidance. *BMJ* 2015;**350**:h1258.

8. Band R, Morton K, Stuart B, et al. Home and Online Management and Evaluation of Blood Pressure (HOME BP) digital intervention for self-management of uncontrolled, essential hypertension: a protocol for the randomised controlled HOME BP trial. *BMJ open* 2016;**6**(11):e012684.

9. Yardley L, Morrison L, Bradbury K, et al. The person-based approach to intervention development: application to digital health-related behavior change interventions. *J Med Internet Res* 2015;**17**(1).

10. Tong A, Sainsbury P, Craig J. Consolidated criteria for reporting qualitative research (COREQ): a 32-item checklist for interviews and focus groups. *Int J Qual Health Care* 2007;**19**(6):349-57.

For peer review only - http://bmjopen.bmj.com/site/about/guidelines.xhtml

11. Band R, Bradbury K, Morton K, et al. Intervention planning for a digital intervention for self-management of hypertension: a theory-, evidence-and person-based approach. *Implementation Science* 2017;**12**(1):25.

12. Braun V, Clarke V. Using thematic analysis in psychology. *Qual Res Psychol* 2006;**3**(2):77-101.

13. Marks DF, Yardley L. *Research methods for clinical and health psychology*: Sage, 2004.

14. Strauss A, Corbin JM. *Grounded theory in practice*: Sage, 1997.

15. Charmaz K. *Constructing grounded theory*: Sage, 2014.

16. NVivo qualitative data analysis Software [program], 2012.

17. Rogers PJ, Stevens K, Boymal J. Qualitative cost-benefit evaluation of complex, emergent programs. *Eval Program Plann* 2009;**32**(1):83-90.

18. Horne R, Weinman J. Patients' beliefs about prescribed medicines and their role in adherence to treatment in chronic physical illness. *J Psychosom Res* 1999;**47**(6):555-67.

19. Mair FS, May CR. Thinking about the burden of treatment. *Br Med J* 2014;**349**:g6680.

20. Leventhal H, Diefenbach M, Leventhal EA. Illness cognition: using common sense to understand treatment adherence and affect cognition interactions. *Cognit Ther Res* 1992;**16**(2):143-63.

21. Eton DT, Yost KJ, Lai J-s, et al. Development and validation of the Patient Experience with Treatment and Self-management (PETS): a patient-reported measure of treatment burden. *Qual Life Res* 2017;**26**(2):489-503.

22. Tran V-T, Harrington M, Montori VM, et al. Adaptation and validation of the Treatment Burden Questionnaire (TBQ) in English using an internet platform. *BMC Med* 2014;**12**(1):109.

23. NICE. Incorporating economic evaluation. 2014. https://www.nice.org.uk/process/pmg20/chapter/incorporating-economic-evaluation.12th July 2017

24. Coast J, Kinghorn P, Mitchell P. The development of capability measures in health economics: opportunities, challenges and progress. *Patient* 2015;**8**(2):119-26.

For peer review only

25. Nussbaum M, Sen A. *The quality of life*: Clarendon Press - Oxford, 1993.

26. Brazier J, Ratcliffe J, Saloman J, et al. *Measuring and valuing health benefits for economic evaluation*: OXFORD university press, 2017.

27. Al-Janabi H, Flynn TN, Coast J. Development of a self-report measure of capability wellbeing for adults: the ICECAP-A. *Qual Life Res* 2012;**21**(1):167-76.

28. Netten A, Burge P, Malley J, et al. Outcomes of social care for adults: developing a preference-weighted measure. *Health Technol Assess* 2012;**16**(16).

29. Ryan M, Kinghorn P, Entwistle VA, et al. Valuing patients' experiences of healthcare processes: Towards broader applications of existing methods. *Soc Sci Med* 2014;**106**:194-203.

30. Donaldson C, Shackley P. Does “process utility” exist? A case study of willingness to pay for laparoscopic cholecystectomy. *Soc Sci Med* 1997;**44**(5):699-707.

31. Bradbury K, Morton K, Grist R, et al. Using the Person-Based Approach to optimise a digital intervention for the management of hypertension. *In submission* 2017.

For peer review only

For peer review only

For peer review only - http://bmjopen.bmj.com/site/about/guidelines.xhtml

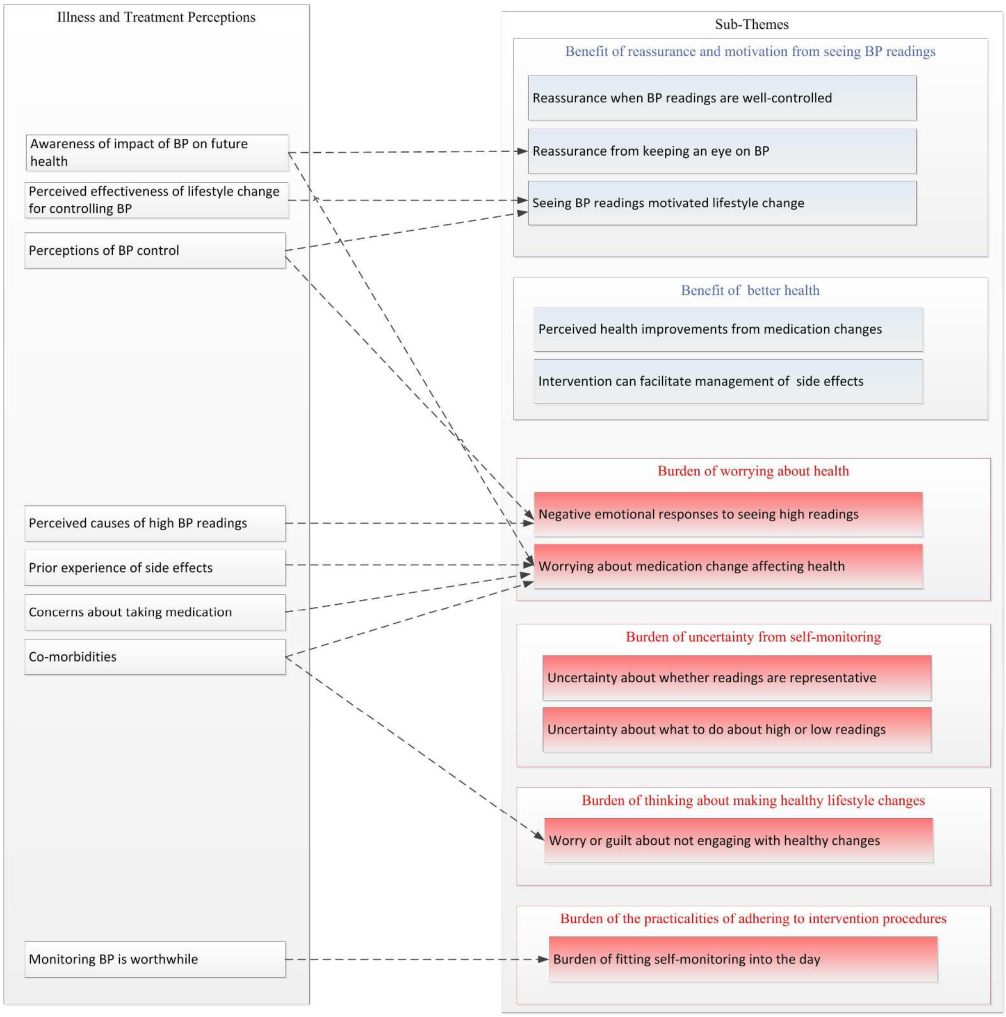

Figure 1. Possible influences of illness and treatment beliefs on perceived burdens and benefits of the intervention

337x340mm (300 x 300 DPI)

Consolidated criteria for reporting qualitative studies (COREQ): 32-item checklist

Developed from:
Tong A, Sainsbury P, Craig J. Consolidated criteria for reporting qualitative research (COREQ): a 32-item checklist for interviews and focus groups. *International Journal for Quality in Health Care*. 2007. Volume 19, Number 6: pp. 349 – 357

YOU MUST PROVIDE A RESPONSE FOR ALL ITEMS. ENTER N/A IF NOT APPLICABLE

No. Item	Guide questions/description	Reported on Page #
**Domain 1: Research team and reflexivity**		
Personal Characteristics		
1. Interviewer/facilitator	Which author/s conducted the interview or focus group?	Page 9
2. Credentials	What were the researcher's credentials? E.g. PhD, MD	Page 9
3. Occupation	What was their occupation at the time of the study?	Page 9
4. Gender	Was the researcher male or female?	Page 9
5. Experience and training	What experience or training did the researcher have?	Page 9
Relationship with participants		
6. Relationship established	Was a relationship established prior to study commencement?	N/A
7. Participant knowledge of the interviewer	What did the participants know about the researcher? e.g. personal goals, reasons for doing the research	N/A
8. Interviewer characteristics	What characteristics were reported about the interviewer/facilitator? e.g. Bias, assumptions, reasons and interests in the research topic	Page 9
**Domain 2: study design**		
Theoretical framework		
9. Methodological orientation and Theory	What methodological orientation was stated to underpin the study? e.g. grounded theory, discourse analysis, ethnography, phenomenology, content analysis	Page 9
Participant selection		
10. Sampling	How were participants selected? e.g. purposive, convenience, consecutive, snowball	Page 8
11. Method of approach	How were participants approached? e.g. face-to-face, telephone, mail, email	Page 8

For peer review only

12. Sample size	How many participants were in the study?	Page 10
13. Non-participation	How many people refused to participate or dropped out? Reasons?	Page 10
Setting		
14. Setting of data collection	Where was the data collected? e.g. home, clinic, workplace	Page 8
15. Presence of non-participants	Was anyone else present besides the participants and researchers?	N/A
16. Description of sample	What are the important characteristics of the sample? e.g. demographic data, date	Page 11
Data collection		
17. Interview guide	Were questions, prompts, guides provided by the authors? Was it pilot tested?	Page 9
18. Repeat interviews	Were repeat inter views carried out? If yes, how many?	N/A
19. Audio/visual recording	Did the research use audio or visual recording to collect the data?	Page 9
20. Field notes	Were field notes made during and/or after the interview or focus group?	Page 9
21. Duration	What was the duration of the inter views or focus group?	Page 11
22. Data saturation	Was data saturation discussed?	Page 9
23. Transcripts returned	Were transcripts returned to participants for comment and/or correction?	N/A
**Domain 3: analysis and findings**		
Data analysis		
24. Number of data coders	How many data coders coded the data?	Page 9
25. Description of the coding tree	Did authors provide a description of the coding tree?	Page 13
26. Derivation of themes	Were themes identified in advance or derived from the data?	Page 9-10
27. Software	What software, if applicable, was used to manage the data?	Page 10
28. Participant checking	Did participants provide feedback on the findings?	Page 26
Reporting		
29. Quotations presented	Were participant quotations presented to illustrate the themes/findings? Was each quotation identified? e.g. participant number	Throughout results
30. Data and findings consistent	Was there consistency between the data presented and the findings?	Yes
31. Clarity of major themes	Were major themes clearly presented in the findings?	Yes
32. Clarity of minor themes	Is there a description of diverse cases or discussion of minor themes?	Yes

For peer review only

Once you have completed this checklist, please save a copy and upload it as part of your submission. When requested to do so as part of the upload process, please select the file type: *Checklist*. You will NOT be able to proceed with submission unless the checklist has been uploaded. Please DO NOT include this checklist as part of the main manuscript document. It must be uploaded as a separate file.

For peer review only

Supplementary File 2. Interview schedules for intervention and usual care group

Intervention group interview schedule

Opening questions

Can you tell me about what it is like for you to have high blood pressure?

How did you feel when you were first told you had high blood pressure?

Can you tell me why you decided to sign up for the Home BP study?

I'm really interested in hearing about your experiences of using HOME BP, can you tell me all about it?

Can you tell me about anything that you have liked about the HOME BP study so far?

Can you tell me about anything that you have disliked?

Can you tell me about any advantages of using HOME BP?

And can you tell me about any disadvantages of using HOME BP?

How do you feel about your high blood pressure now?

Do you think you would choose to keep on using the HOME BP programme if it was still available after a year?

Why/ Why not?

Would you recommend the HOME BP programme to other people with high blood pressure?

Self-monitoring

Experiences of monitoring:

Can you tell me about what it was like when you were learning how to use your BP monitor at home?

Can you tell me about the first time you used your BP monitor at home in the study?

For peer review only

Can you tell me about what it has been like to monitor your blood pressure yourself?

How did it make you feel?

Had you ever monitored your own blood pressure before you took part in the HOME BP study?

If yes:

Can you tell me a bit more about this?

Can you tell me whether you've noticed any changes in how you monitor your blood pressure since you've started the HOME BP programme?

All: Can you tell me about any concerns that you have about monitoring your blood pressure?

<u>Adherence to the monitoring schedule:</u>

The HOME BP programme reminds you to monitor your blood pressure for 7 days every 4 weeks.

Can you tell me about what it has been like for you to try and monitor your blood pressure for 7 days every 4 weeks?

Can you tell me about the time of day you have been monitoring your blood pressure so far?

How do you feel about the instructions HOME BP gives about when to monitor?

Was there anything that helped you to monitor your own blood pressure?

Was there anything that made it harder to monitor your own blood pressure?

Can you tell me about any time when you had to skip your blood pressure monitoring or change when you did it in the day?

Can you tell me how you found it entering your readings on the HOME BP programme?

For peer review only

Feedback messages

Remember after you enter your readings on HOME BP, you see a message which says 'your readings were amber, or green'? What did you think about these messages?

Can you tell me about how you felt when you saw a message about your blood pressure readings on HOME BP?

How did you feel about monitoring your own blood pressure after reading that message?

Did you have any very high or very low readings (red/blue) when you were monitoring in the HOME BP study? How did you feel?

2. Medication change

Can you tell me about what it's like for you taking medication for your high blood pressure?

Have you been recommended to make a medication change during the HOME BP study?

If yes:

How did you feel about making a change to your medication when HOME BP recommended it?

Did you make this change to your medication?

If they chose to make a change:

Can you tell me about what it was like to make a medication change in the HOME BP study?

Can you tell me about how the medication change came about?

How did you get on with your new medication?

How would you feel if HOME BP recommended a medication change for you now?

For peer review only

If they chose not to make a change –

Can you tell me about why you decided not to change your medication?

How would you feel if HOME BP recommended another medication change for you now?

If no:

If patient has ***not*** *been recommended a med change:* How do you feel about changing your medication if your blood pressure stayed too high for too long?

Can you tell me about any concerns that you have about doing this?

All:

How did you feel about making changes to your medication before you took part in HOME BP?

3. HCP support

Can you tell me about the contact you have had with your GP or nurse so far in the HOME BP study?

Can you tell me about how you have found the support from your GP or nurse while you were taking part in the HOME BP study?

Can you tell me what you thought about receiving emails from your nurse about HOME BP?

If needed, follow-up questions:

Have you seen your GP about your blood pressure since the start of the study?

And what about appointments with the nurse?

Can you tell me about how you have found the emails from HOME BP?

4. Healthy changes

Can you tell me about anything else that you do to try and manage your high blood pressure?

For peer review only

There is an option on the HOME BP programme to look at online sessions about making healthy changes that can help lower your blood pressure, I wonder if you remember if you have seen it?

If no:

Can you tell about how you might feel about making healthy changes to help lower your blood pressure?

If yes:

Can you tell me about how you found the session on healthy changes?

How did you feel about making the healthy changes this session talked about?

If they chose a change: Can you tell me about what made you choose this/these ones?

How did you find the online session about xxxx (cutting down on salt/alcohol/eating a healthier diet/doing more physical activity/ losing weight)?

Can you tell me about what it was like for you trying out this healthy change?

How did making a healthy change like this make you feel?

Or if they didn't choose any: Can you tell me about why you didn't want to choose a healthy change?

All: Can you tell me about any concerns that you have about making a new healthy change?

Is there anything else you would like to tell me about the HOME BP study that we haven't already talked about?

For peer review only

Usual care group interview schedule

General questions about BP

Can you tell me about what it is like for you to have high blood pressure?

How did you feel when you were first told you had high blood pressure?

Can you talk me through what (if anything) you currently do to manage/control your high blood pressure?

Can you tell me about how your GP and practice nurse are involved in helping you to control your high blood pressure?

How do you feel about monitoring your own blood pressure at home?

If they already monitor – can you tell me a bit more about this?

Can you tell me about what it's like for you taking medication for your high blood pressure?

Can you tell me how you feel about your GP making changes to your blood pressure medication?

HOME BP study experience

Can you tell me why you signed up for the Home BP study?

Can you tell me all about what it has been like for you being in the HOME BP study in the usual care group?

At the start of the HOME BP study, you had a baseline appointment with a nurse at your GP Practice – can you tell me about that?

How did you find it using the HOME BP programme to fill in your questionnaires online at the start of the study?

For peer review only

After you had completed your questionnaires, did you have an appointment with your GP to check your blood pressure medication? Can you tell me about this?

<u>HCP support</u>

Can you tell me about the contact you have had with your GP or nurse so far in the HOME BP study?

Can you tell me about how you have found the support from your GP or nurse while you were taking part in the HOME BP study?

For peer review only
